# Treatment of Men for “Low Testosterone”: A Systematic Review

**DOI:** 10.1371/journal.pone.0162480

**Published:** 2016-09-21

**Authors:** Samantha Huo, Anthony R. Scialli, Sean McGarvey, Elizabeth Hill, Buğra Tügertimur, Alycia Hogenmiller, Alessandra I. Hirsch, Adriane Fugh-Berman

**Affiliations:** 1 Tulane University, School of Medicine, New Orleans, LA, United States of America; 2 Department of Pharmacology and Physiology, Georgetown University Medical Center, Washington, DC, United States of America; 3 Scialli Consulting LLC, Washington, DC, United States of America; 4 University of South Florida, Tampa, FL, United States of America; 5 University of Illinois, Chicago, IL, United States of America; University of Kansas Medical Center, UNITED STATES

## Abstract

Testosterone products are recommended by some prescribers in response to a diagnosis or presumption of “low testosterone” (low-T) for cardiovascular health, sexual function, muscle weakness or wasting, mood and behavior, and cognition. We performed a systematic review of 156 eligible randomized controlled trials in which testosterone was compared to placebo for one or more of these conditions. We included studies in bibliographic databases between January 1, 1950 and April 9, 2016, and excluded studies involving bodybuilding, contraceptive effectiveness, or treatment of any condition in women or children. Studies with multiple relevant endpoints were included in all relevant tables. Testosterone supplementation did not show consistent benefit for cardiovascular risk, sexual function, mood and behavior, or cognition. Studies that examined clinical cardiovascular endpoints have not favored testosterone therapy over placebo. Testosterone is ineffective in treating erectile dysfunction and controlled trials did not show a consistent effect on libido. Testosterone supplementation consistently increased muscle strength but did not have beneficial effects on physical function. Most studies on mood-related endpoints found no beneficial effect of testosterone treatment on personality, psychological well-being, or mood. The prescription of testosterone supplementation for low-T for cardiovascular health, sexual function, physical function, mood, or cognitive function is without support from randomized clinical trials.

## 1. Introduction

Testosterone and methyltestosterone are marketed in the United States for men with congenital or acquired hypogonadism. Some practitioners have used testosterone preparations to treat a variety of symptoms identified as those of “low testosterone” (low-T), a term that has not been uniformly defined. We present a systematic review of randomized controlled trials (RCTs) that evaluated the use of testosterone therapy against placebo or inactive comparator in adult men for cardiovascular health, sexual function, muscle weakness/wasting, mood and behavior, or cognition. We did not include studies of testosterone in men with missing or damaged testicles, or who had Klinefelter syndrome or other genetic anomalies. We did not include studies on the use of testosterone for any indication in women or in children, the use of androgens in contraception, or the use of androgens for bodybuilding or athletic performance.

## 2. Methods

### 2.1 Data Search, Synthesis and Analysis

Computerized literature searches were conducted in PubMed, Embase, and APA PsycNET. Searches were limited to human males but were not restricted by language or date. The PubMed search was conducted using the MeSH term “testosterone” and the modifiers “administration and dosage,” “adverse effects,” “deficiency,” “standards,” “therapeutic use,” or “therapy.” The original PubMed search was conducted for studies published between January 1, 1950 and November 26, 2013. The Embase search was conducted using the Emtree key term “testosterone,” modified by “adverse drug reaction,” “androgen deficiency,” “therapy,” “drug dose,” or “clinical trial.” The original Embase search was conducted for studies published between January 1, 1974 and November 26, 2013. The PsycNET search was conducted using the term “testosterone” modified by “addiction,” “drug dependency,” “therapy,” “treatment,” or “deficiency.” The original PsycNET search was conducted for studies published between January 1, 1806 and November 26, 2013. All searches were repeated on April 9, 2016 to identify clinical trials that had been published since the initial search, so the final search included more than four decades of trials from all databases.

### 2.2 Study Selection

Search results were combined using EndNote and duplicates were deleted. These results were filtered using the key term “clinical trial.” Titles and abstracts were reviewed to identify RCTs and eliminate irrelevant studies. Relevant studies were retrieved.

### 2.3 Data Extraction

Data were extracted into tables by 4 independent reviewers according to the presence of information on cardiovascular health, sexual function, muscle weakness/wasting, mood and behavior, or cognition. Studies with multiple relevant endpoints were included in all relevant tables. Review articles were identified and retrieved, and their reference lists were searched for primary publications of RCTs.

Some studies that included randomized controlled designs also included open-label continuation phases. We evaluated and summarized the randomized controlled portions of these studies. Although our primary interest was the use of testosterone for the treatment of hypogonadism, however defined by study authors, we included trials of testosterone in eugonadal men. In some studies, eugonadal subjects were randomized to receive testosterone or a comparator (usually placebo), and hypogonadal subjects were treated with testosterone only. We evaluated and summarized only the randomized portions of these studies.

### 2.4 Quality Assessment

We assessed quality of studies by a 5-point Jadad score. In order to be as inclusive as possible, we included all studies identified regardless of Jadad score. For clinical endpoints only (angina/ischemia, congestive heart failure, and erectile dysfunction) we also included an analysis of studies restricted to Jadad scores of 4 or 5. We accepted whatever criteria were used by individual study authors to define low testosterone.

## 3. Results

**Fig 1:**
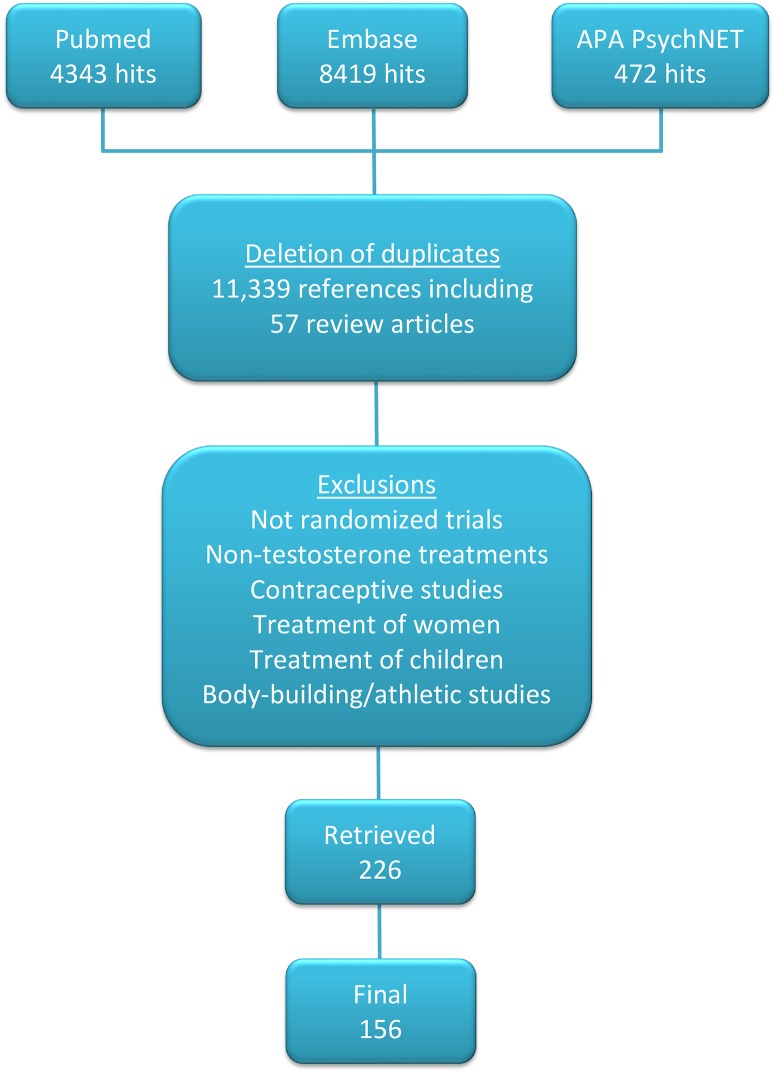
Literature Search and Study Selection.

Fig 1 lists the exclusion criteria used to select 226 qualifying papers from 11,417 reviewed abstracts. Although most studies were described by their authors as randomized, not all indicated the nature of the randomization procedures. Some studies included identical numbers of subjects in treatment and exposed conditions, suggesting that allocation was not random. After further examination, 70 papers did not meet our criteria, so the final data set included 156 papers.

### 3.1 Cardiovascular Health

Table 1 summarizes extracted studies that focused on the effect of testosterone on cardiovascular endpoints, including 17 studies on ischemia/angina, 6 on congestive heart failure (CHF), 25 on lipids, and 11 on inflammatory and coagulation markers.

**Table 1:** Effects of Testosterone on Cardiovascular Endpoints.

**Ischemia/Angina**				
***Acute Intravenous Treatment***				
**Rosano et al, 1999**[12]	14 men 45–66 years old with coronary artery disease (CAD).	2.5 mg testosterone (T) or intravenous (IV) placebo over 5 min, 30 min prior to exercise test; tx switch at 2 days; randomization by computer; masking not described.	↑Time to ST segment depression. ↓Maximum ST segment depression. ↓ST recovery.	3
**Ong et al, 2000**[9]	22 men with CAD.	2.3 mg T or placebo IV over 10 min (n = 11) with switch after 1 week; 0.023–0.046 mg T or placebo IV over 10 min (n = 11) with tx switch after 1 week; randomization method not given.	↑Percent change in brachial artery diameter after release of occlusion with high-dose T. No change in flow velocity in brachial artery after release of occlusion. Low-dose T had no effect. Interpreted as enhanced response to local effects of nitric oxide after T.	3
**Thompson et al, 2002**[8]	34 men 69 ± 6 years old (mean ± SD) with CAD and exercise- or adenosine-inducible ischemia.	T or placebo by bolus IV over 20 min with maintenance IV to increase basal serum T concentration 0, 2-, or 6-fold with each subject receiving all 3 conditions randomly 1 week apart; randomization method not given.	No effect on time to ST segment depression or myocardial perfusion defects by SPECT. No effect on time to angina in the 5 subjects who experienced angina during testing.	4
**Webb et al, 1999**[2]	14 men 35–75 years old with CAD and plasma T concentration ≤ 11 nM (317 ng/dL).	2.3 mg T or placebo IV over 10 min 30 min prior to exercise test; 1 week later under tx switch; randomization method not given.	↑Time to ST segment depression. No change in maximum ST segment depression or in time to onset of angina.	4
**2–24 Week Treatment Period**				
**Dohn et al, 1968**[13]	44 men with leg claudication or ulcers attributed to arteriosclerosis (n = 43) or Buerger’s diseas (n = 1). Two men did not complete study, but numbers in tables add to 86 subjects. Not possible to tell for sure how many men were analyzed.	300 mg aqueous T isobutyrate or placebo (meprobamate) every 14 days for 3 months; route of administration not given; double-blind; randomization method not given.	No effect on subject improvement, walking test, plethysmographic estimation of pulse volume, blood flow at rest or after exercise, or hyperemia after compression. ↑Skin temperature.	3
**Ly et al, 2001**[11]	37 men mean age 68.2 years with plasma T concentration ≤15 nM (432 ng/dL); 4 dropouts were excluded from analysis.	70 mg dihydrotestosterone (DHT) gel (n = 18) or placebo (n = 19) applied daily for 3 months; randomization method not given.	No effect on flow- or nitroglycerin-mediated dilatation of brachial artery.	5
**Kang et al, 2002**[10]	35 men mean age 58 years with CAD.	160 mg testosterone undecanoate PO daily for 4 weeks then 80 mg daily for 8 weeks (n = 18); placebo tx (n = 17) not given; randomization method not given.	↑Flow- and nitroglycerin-mediated dilatation of brachial artery.	1
**Malkin et al, 2004**[1]	12 men 60.8 ± 4.6 years old (mean ± SD) with CAD and “clinical need for T replacement.” One man failed screening and another withdrew at unspecified point in the study.	100 mg T or placebo IM every 2 weeks for 4 weeks, 1 month washout, then tx switch; randomization by computer; single-blind.	↑Time to ST segment depression. No significant change in Seattle Angina Score. ↓Beck Depression Inventory (BDI) score.	5
**Jaffe, 1977**[14]	50 men 35–71 years old with post-exercise ST segment depression.	200 mg T cypionate (n = 25) or placebo IM (n = 25) weekly for 8 weeks, randomization method not given. Described as double-blinded.	↓Sum of ST segment depression in leads II, V4, 5, and 6 at 0, 2, 4, and 6 min after exercise. Symptoms not evaluated.	4
**Cornoldi et al, 2009**[15]	87 men 57–74 years old with chronic angina, CAD, or prior myocardial infarction (MI).	40 mg T undecanoate (n = 43) or placebo (n = 44) PO TID for 12 weeks; double-blind; randomization by computer.	↓Incidence of silent myocardial infarction. ↓Total ischemic burden. ↓Number of anginal attacks/week.	5
**English et al, 2000**[16]	50 men mean age 62 years with stable CAD.	5 mg daily T patch (n = 25) or placebo (n = 25) for 12 weeks; double-blind; randomization method not given. Three withdrawals from T arm and 1 withdrawal from placebo arm eliminated from analysis.	↑Time to ST-segment depression by week 14. No change in angina frequency. Improved quality of life (QoL) scores.	4
**Webb et al, 2008**[3]	25 men 40–75 years old with angiographically proven CAD (≥70% lesion in at least one major coronary artery or major branch), plasma T concentration ≤12 nM (346 ng/dL); 2 dropouts prior to medication tx were not analyzed. One subject had unanalyzable data.	160 mg/day T undecanoate or placebo PO for 8 weeks followed by tx switch. Method of randomization not given.	No change in global myocardial perfusion by magnetic resonance imaging (MRI). ↑Perfusion of segments supplied by coronary arteries without significant obstruction. ↑Left ventricular (LV) ejection fraction (EF) (by 2%). No change in stroke volume (SV), end-systolic volume (ESV), end-diastolic volume (EDV), or heart mass.	4
**Basaria et al, 2010**[17]	209 men ≥65 years old with total serum T 100–350 ng/dL (3.5–12.1 nM) or free serum T <50 pg/mL (174 pM) and with mobility limitations. Analysis restricted to 176 men with a baseline assessment and at least one outcome assessment.	100 mg T (n = 106) or placebo (n = 103) gel daily for 6 months. After 2 weeks, dose level was increased or decreased by 50% based on serum T. Randomization was by age blocks but was not otherwise described.	↑Cardiovascular-related adverse events (AEs), adjusted OR 5.8, 95% CI 2.0–16.8 (includes acute coronary syndrome [ACS]–chest pain, syncope, MI, stroke, congestive heart failure [CHF] exacerbation, coronary stenting and bypass procedures, peripheral edema, elevated blood pressure [BP], arrhythmias, ECG changes).	5
**Wu et al, 1993**[7]	62 men 55–75 years old with angina.	120 mg T undecanoate PO QD × 2 weeks then 40 mg/day × 2 weeks or placebo for 2 weeks followed by 2-week washout, then tx switch; described as randomized, but there were 31 men in each group and randomization was not described.	↓Ischemia on ECG and Holter recordings by subjective scoring system. ↓Angina by subjective scoring system.	2
**≥12-Month Treatment Period**				
**Mathur et al, 2009**[4]	15 men 64.8 ± 7 years old (mean ± SD) with stable chronic angina, ST segment depression at baseline, and at least 2 early morning serum T concentrations < 12 nM (346 ng/dL); one man assigned to each arm withdrew at unspecified point in the study.	1000 mg T undecanoate depot IM (n = 6) or placebo (n = 7) q 3 months × 12 months; randomized by computer; described as double-blind.	↑Time to ST segment depression at 14, 28, and 52 weeks and increased level of exercise attained. No significant change in Seattle Angina Score (SAS).	4
**Kenny et al, 2002** [5]	67 men 65–87 years old (mean 74) with bioavailable T <4.44 nM (128 ng/dL); 23 dropouts (10 T, 13 placebo) and 8 men with technical difficulties were not included in the analysis.	5 mg daily T patch (n = 34) or placebo (n = 33) × 12 months. Randomization method not described.	No change in vascular reactivity after occlusion.	3
**Basaria et al, 2015**[6]	Men aged 60 years or older, morning total T 100–400 ng/dL (3.5–14 nM) or free <50 testosterone pg/mL (1.7 pM). 1:1 concealed randomization with stratification by age dichotomized at 75 years and by site. Computer-generated randomization. All subjects receiving at least 1 medication dose were retained for analysis.	3 years of daily application of 75 (n = 155) or 0 (n = 151) mg testosterone as a gel, dose level adjusted upwards or downwards based on total testosterone 2–12 hours after gel application. Placebo adjusted by an unblinded observer. 44/155 receiving T did not complete, 23 for adverse events; 51/151 receiving placebo did not complete, 17 for adverse events.	No difference in carotid artery intima-media thickness or in rate of thickening over time; No difference in coronary artery calcium score change over time	5
**Congestive Heart Failure**				
**Caminiti et al, 2009**[18]; **Schwartz et al, 2011**[87]	70 men 66–76 years old (mean age 70) with stable CHF (NYHA II or III) and LV E_f_ <40%. Dropouts (4 on T and 2 on placebo) and lost data (1 on T and 5 on placebo) were not included in analysis.	1000 mg T undecanoate (n = 35) or saline (n = 35) IM at 6 and 12 weeks. Subjects said to be randomized, but randomization method was not given.	↑Distance in 6-minute walk test. ↑Body mass index (BMI). ↑O_2_ consumption. ↓Ventilation/CO_2_ output. ↓Diastolic BP. No change in EF or LV end-diastolic diameter.[18] ↓QT interval (0.8%) and ↓QT_c_ interval (1.5%).[87]	4
**Pugh et al, 2003**[19]	12 men 48–82 years old with stable CHF; 8 men characterized as having ischemic heart disease, 2 had dilated cardiomyopathy, 1 had hypertension, and 1 had alcohol-related heart failure.	60 mg T or placebo given buccally followed the next day by tx switch. Described as randomized, but randomization method not given.	Cardiac index was positively correlated and systemic vascular resistance negatively correlated with serum free T concentration. T attenuated the fall in cardiac index and the rise in systemic vascular resistance associated with the catheterization procedure. No effect of tx on pulmonary capillary wedge pressure. Another report of this trial found no effect on serum concentration of TNF-α.[21]	3
**Pugh et al, 2004**[20]	20 men 44–81 years old with impaired LV EF (mean 35%).	100 mg T or placebo IM every 2 weeks for 12 weeks. Subjects said to be randomized, but randomization method and number of subjects per group not given.	Distance walked was increased more by T than by placebo. T improved heart failure symptom scores compared to baseline. There was no improvement after placebo. There were no effects of T on LV size, or EF.	3
**Malkin et al, 2006**[23]	76 men with CHF, mean age ~64 years; 34 dropouts were retained for analysis using ITT.	5 mg T (n = 37) or placebo (n = 39) patch daily for 12 months; randomization stratified by ischemic vs non-ischemic heart failure. Method of randomization not given.	15% improvement in distance on shuttle walk test. More subjects on T (35%) than placebo (8%) improved in NYHA class.	3
**Mirdamadi et al 2014**[22]	50 males, age 50–70, with CHF.	T enanthate 250 mg IM or saline placebo IM every 4 weeks for 12 weeks.	No difference between groups in blood pressure (SBP or DBP), ejection fraction, or other cardiovascular end points assessed by echocardiography. A Doppler-based myocardial performance index improved in the treatment group.	3
**Lipids**				
***Favorable Effects on Lipids***				
**Uyanik et al, 1997** [155]	37 healthy men 53–89 years old.	120 mg daily T undecanoate (n = 17) or placebo (n = 20) PO for 2 months.	↓Total serum cholesterol (12%). ↓LDL cholesterol (20.7%). No change in HDL cholesterol or triglycerides.	0
**Tenover, 1992**[156]	13 healthy men 57–76 years old with serum T ≤13.9 nM (400 ng/dL).	100 mg T enanthate or placebo IM weekly x 3 months followed by tx switch x 3 months; described as randomized, but randomization procedure not described. Six subjects received T first.	↓Total serum cholesterol (11%). ↓LDL cholesterol (12%). No effect on HDL cholesterol, apolipoprotein A-1, or triglycerides.	4
**Ly et al, 2001**[11]	37 men mean age 68.2 years with plasma T concentration ≤15 nM (432 ng/dL); 4 dropouts were excluded from analysis.	70 mg dihydrotestosterone (DHT) gel (n = 18) or placebo (n = 19) applied daily for 3 months; method of randomization not discussed.	↓Total serum cholesterol (~10%). ↓LDL cholesterol (~10%). No change in HDL cholesterol or triglycerides.	5
**Howell et al, 2001**[24]	35 men, mean age 40.9 years after cancer chemotherapy; serum luteinizing hormone ≥8 IU/L and serum T < 20 nM (576 ng/dL); 2 subjects did not complete the study; it is not known if they were included in analysis.	2.5 mg T (n = 16) or placebo (n = 19) patch daily for 2–4 weeks then dose increased to 2 patches daily for remainder of 12 months unless serum T >20 nM; randomization method not given.	↓LDL cholesterol (13%) for periodic measurements averaged over months 3–12. No change in triglycerides, LDL cholesterol, HDL cholesterol.	3
**Malkin et al, 2004**[27]	29 men 36–78 years old with a clinical indication for T replacement for hypogonadism; 2 subjects were withdrawn and 2 additional patients did not contribute analyzable sera.	100 mg T (n = 27) or placebo IM (n = 27) every 2 weeks; randomization using blocks of computer-generated numbers. A crossover design appears likely, although not explicit.	↓Total serum cholesterol (6%). ↓Triglycerides (11%). No effect on LDL or HDL cholesterol.	5
**Malkin et al, 2004**[1]	12 men 60.8 ± 4.6 years old (mean ± SD) with CAD and “clinical need for T replacement”; one man failed screening, one man withdrew at unspecified point in the study.	100 mg T or placebo IM every 2 weeks for 4 weeks, washout for 1 month, then opposite tx; order of tx randomized by computer; described as single-blinded.	↓Total serum cholesterol (6%). No effect on LDL or HDL cholesterol or triglycerides.	5
**Kapoor et al, 2006**[157]	27 men, 52–76 years old (mean age 54 years) with type 2 diabetes mellitus (T2DM) and total T <12 nM (346 ng/dL) with symptoms attributed to hypogonadism; 3 men were excluded due to protocol violations.	200 mg sustanon (30 mg T propionate, 60 mg T phenylpropionate, 60 mg T isocaproate, and 100 mg/mL T decanoate) or placebo IM every 2 weeks for 6 injections followed by a 1-month washout period followed by tx switch; randomization by computer-derived random number table; number in each arm not stated.	↓Total serum cholesterol (5%). No effect on LDL or HDL cholesterol or triglycerides.	5
**Mathur et al, 2009**[4]	15 men 64.8 ± 7 years old (mean ± SD) with stable chronic angina, ST segment depression at baseline, and at least 2 early morning serum T concentrations < 12 nM (346 ng/dL); one man assigned to each arm withdrew at unspecified point in the study.	1000 mg T undecanoate depot IM (n = 6) or placebo (n = 7) q 3 months for 12 months; randomized by computer; described as double-blind.	↓Triglycerides (% change not available). No effect on total or HDL cholesterol.	4
**Cornoldi et al, 2010**[15]	87 men 57–74 years old with chronic angina, CAD, or prior MI.	40 mg T undecanoate (n = 43) or placebo (n = 44) PO TID for 12 weeks; double-blind; randomization by computer.	↓Serum total cholesterol (7%). ↓Triglycerides (14%). No effect on HDL cholesterol.	5
**Gianatti et al 2014**[32]	88 men age 35–70 years of age with a history of type 2 diabetes mellitus (T2DM) and total testosterone ≤12.0 nM (346 ng/dL). 13 men did not complete the study, 8 because of intensification of oral hypoglycemic agents or commencement of insulin therapy. 1 subject in testosterone group was withdrawn with a hematocrit of >54 prior to his 30 week injection.	Participants were randomly assigned in a concealed 1:1 allocation to T or placebo using permuted blocks with a block size of 4. IM T undecanoate 1000 mg (n = 45) or placebo (n = 43) at 0, 6, 18, and 30 weeks.	↓Total cholesterol (12%); ↓ LDL cholesterol (13%); ↑ HDL cholesterol (9%); No change in triglycerides	4
**Hackett et al 2014**[158]	199 Men aged 18–80 with T2D with a total T 8.1–12 nM (234–346 ng/dL) or total T of ≤8.0 nM (231 ng/dL). 9 patients did not complete the study; 4 because of serious adverse events (3 treatment unrelated deaths, 1 prostate cancer in placebo) and 5 withdrew their consent.	Subjects were block randomized to receive T undecanoate IM at 0 (n = 102) or 1000 (n = 97) mg at week 0, 6, and 18.	↓Total cholesterol (6%); No change in LDL or HDL cholesterol or triglycerides	5
***Lack of Favorable Effects on Lipids***				
**Kang et al, 2002**[10]	35 men mean age 58 years with CAD.	160 mg T undecanoate PO daily for 4 weeks then 80 mg daily for 8 weeks (n = 18). Placebo tx (n = 17) not described. Randomization method not given.	No effect on total HDL and LDL cholesterol or on triglyceride serum concentration.	1
**Kenny et al, 2002**[5]	67 men 65–87 years old (mean 74) with bioavailable T <4.44 nM (128 ng/dL); 23 dropouts (10 T, 13 placebo) not included in the analysis.	5 mg daily T patch (n = 34) or placebo (n = 33) for 1 year. Randomization method not described.	↓HDL (9%) and HDL_2_ (15%) cholesterol. No change in total, LDL cholesterol, triglycerides, or lipoprotein-a (LP-a).	3
**Chung et al, 2007**[61]	30 healthy men 18–45 years old.	200 mg T (n = 10), nonandrolone (n = 10), or placebo IM (n = 10) weekly for 4 weeks; computer-generated randomization list with block design.	No effect on total, LDL, or HDL cholesterol or triglyceride serum concentrations.	5
**Kouri et al, 1996**[159]	16 healthy men 20–43 years old.	T cypionate IM (150 mg at weeks 1 and 2 [n = 8], 300 mg at weeks 3 and 4 [n = 8], and 600 mg at weeks 5 and 6 [n = 8]) or placebo followed by 6-week washout followed by opposite tx and another 6-week washout. Described as randomized, procedure not given.	↓HDL cholesterol (21%). No effect on LDL cholesterol.	3
**Jockenhövel et al, 1999**[25]	55 men with hypogonadism (serum T concentration <3.6 nM [105 ng/dL]); androgen therapy withdrawn 3 months prior to study in men using such therapy.	Randomized by unspecified method to mesterolone (n = 12; not further discussed here), T undecanoate 160 mg/day PO (n = 13), T enanthate (n = 15) 250 mg IM every 21 days, T subcutaneous pellet 200 mg implanted once (n = 15). Open label.	↑Total serum cholesterol (6–20%). ↑LDL cholesterol (47–65%). ↓HDL cholesterol (33–36%). ↑Triglycerides (23–46%).	1
**Snyder et al, 2001**[160]	108 healthy men over 65 years old (mean age 73 years) with serum T concentration at least 1 standard deviation below the mean for young men (16.5 nM [476 ng/dL]).	6 mg daily scrotal T patch (n = 54) or placebo (n = 54); dose could be reduced to 4 mg daily if serum T >1000 ng/dL (34.7 nM). Study described as randomized and double-blinded; randomization method not discussed.	No effect on serum concentrations of total, HDL, or LDL cholesterol, triglycerides, apolipoprotein B, apolipoprotein A-1, or LP-a.	4
**Webb et al, 2008**[3]	25 men 40–75 years old with angiographically proven coronary heart disease (≥70% lesion in at least one major coronary artery or major branch), plasma T concentration ≤12 nM (346 ng/dL); 2 dropouts prior to medication tx were not analyzed. One subject had unanalyzable data.	160 mg/day T undecanoate or placebo by mouth for 8 weeks followed by crossover to the other tx. Allocation of tx order not discussed.	↓HDL cholesterol (9%). No change in total or LDL cholesterol or triglycerides.	4
**Agledahl et al, 2008**[161]	27 men, average age 69 years and serum T <11.0 nM (317 ng/dL); 1 dropout excluded from analysis.	1000 mg T undecanoate (n = 14) or placebo (n = 13) IM at 0, 6, 16, 28, and 40 weeks; randomization method not discussed.	No effect at 52 weeks on postprandial serum triglycerides, chylomicron triglycerides, free fatty acids, lipoprotein lipase, or hepatic lipase after a fatty meal.	2
**Emmelot-Vonk et al, 2008**[91]	237 healthy men 60–80 years old with T concentration below the median; ie, <13.7 nM (395 ng/dL); 30 dropouts, 16 of whom provided some follow-up information.	160 mg T undecanoate (n = 120) or placebo (n = 117) by mouth daily for 6 months; randomization by computer-generated list using blocks of 6.	No effect on total, HDL, or LDL cholesterol or triglycerides.	5
**Kalinchenko et al, 2010**[162]	184 men, 35–69 years old, with metabolic syndrome and T concentration <12.0 nM (346 ng/dL); 14 dropouts were eliminated from analysis.	1000 mg T undecanoate (n = 113) or placebo (n = 71) IM at 0, 6, and 18 weeks; randomization method not discussed, tx arms were intentionally uneven.	No difference in total, HDL, or LDL cholesterol or in triglycerides.	4
**Jones et al, 2011**[26]	220 men, mean age 59.9 years, with metabolic syndrome or T2DM or both and total T ≤11 mM (317 mg/dL) or free T <255 pM (7.3 ng/dL); 54% of subjects completed the study, ITT analysis used last observation carried forward.	60 mg T (n = 108) or placebo (n = 112) gel for 12 months; randomization stratified by presence of metabolic syndrome only, diabetes mellitus (DM) only, and DM with metabolic syndrome; dose levels adjusted based on T measurements.	↓LP-a (23–27%) at months 6 and 9; no difference at month 12; No difference in total, HDL, or LDL cholesterol or in triglycerides	4
**Paduch et al, 2015**[78]	Sexually active men 26 or more years old with ejaculatory dysfunction and total T < 300 ng/dL (10.41 nM).	T solution applied to axilla daily at 60 (n = 39) or 0 (n = 35) mg/day, titrated up or down based on serum T concentration after 4 weeks. Computer randomization scheme on a 1:1 basis. Five subjects in each group discontinued, 1 in T group due to adverse event.	No differences in total, LDL, or HDL cholesterol or triglycerides	5
**Basaria et al, 2015**[6]	Men aged 60 years or older, morning total T 100–400 ng/dL (3.5–14 nM) or free <50 testosterone pg/mL (1.7 pM). 1:1 concealed randomization with stratification by age dichotomized at 75 years and by site. Computer-generated randomization. All subjects receiving at least 1 medication dose were retained for analysis.	3 years of daily application of 75 (n = 155) or 0 (n = 151) mg testosterone as a gel, dose level adjusted upwards or downwards based on total testosterone 2–12 hours after gel application. Placebo adjusted by an unblinded observer. 44/155 randomized to T did not complete, 23 for adverse events; 51/151 receiving placebo did not complete, 17 for adverse events.	No differences in total, LDL, or HDL cholesterol or triglycerides	5
**Asih et al, 2015**[163]	50 men ≥50 years old complaining of memory problems (44 completed) Randomization by random numbers table.	T transdermal 50 (n = 22) or 0 (n-22) applied to the scrotum daily for 24 weeks; after a 4-week washout, patients were crossed over to the other arm	No differences in total, HDL, or LDL cholesterol.	5
**Inflammatoryand Coagulation Markers**				
**Ng et al, 2002**[33]	37 healthy men >60 years of age with serum T concentration <15 nM (432 ng/dL); 4 dropouts were excluded from analysis.	DHT gel 70 mg/day (n = 18) or placebo (n = 19) for 3 months.	No effect on C-reactive protein, soluble intracellular adhesion molecule-1, or soluble vascular cell adhesion molecule-1.	4
**Malkin et al, 2004**[27]	29 men 36–78 years old with a clinical indication for T replacement for hypogonadism; 2 subjects were withdrawn and 2 additional patients did not contribute analyzable sera.	100 mg T (n = 27) or placebo IM (n = 27) every 2 weeks; randomization using blocks of computer-generated numbers. A crossover design appears likely, although not explicit.	↓Serum tumor necrosis factor-α (TNF-α). ↑Interleukin-10 (IL-10). No change in IL-1β (identified as decreased by authors, but not statistically significant).	5
**Malkin et al, 2004**[1]	12 men 60.8 ± 4.6 years old (mean ± SD) with CAD and “clinical need for T replacement”; one man failed screening, one man withdrew at unspecified point in the study.[19]	100 mg T or placebo IM every 2 weeks x 4 weeks, 1 month washout, then tx switch; randomization by computer; described as single-blinded.	↓Serum TNF-α.	5
**Smith et al, 2005**[34]	61 men with CAD recruited, 50 completed screening and placebo run-in phase. Four subjects withdrew and were excluded from analysis.	5 mg T or placebo patches applied each night.	No change in plasma fibrinogen, plasminogen activator inhibitor-1, or tissue plasminogen activator at 6 or 14 weeks tx.	3
**Pugh et al, 2005**[21]	12 men 48–82 years old with stable CHF (same group reported on in 2003)[19]	60 mg T or placebo given buccally followed the next day by tx switch. Described as randomized, but randomization method not given.	No effect on serum concentration of TNF-α with any of these T tx.	3
	20 men with NY Heart Association class II or III CHF, mean age 63.9 years in the active group and 61.1 years in the placebo group.	100 mg T (n = 10) or placebo (n = 10) IM every 2 weeks for 12 weeks. Subjects said to be randomized, randomization method not given.		3
	62 men with NY Heart Association class II, III, or IV CHF, mean age 63.1 years in the active group and 64.9 years in the placebo group.	5 mg T (n = 37) or placebo (n = 39) patch applied daily for 12 weeks. Randomization method not given.		3
**Kapoor et al, 2007**[31]	20 men, 52–76 years old (mean age 63 years) with T2DM and total T <12 nM (346 ng/dL) or bioavailable T <4 nM (115 ng/dL) with symptoms attributed to hypogonadism; 4 men were excluded due to technical problems with measurement.	200 mg Sustanon (30 mg T propionate, 60 mg T phenylpropionate, 60 mg T isocaproate, and 100 mg/mL T decanoate) or placebo IM every 2 weeks for 6 injections followed by a 1-month washout followed by tx switch; tx order randomized by computer-derived random number table; number in each arm not stated.	No effect on C-reactive protein.	5
**Webb et al, 2008**[3]	25 men age 40–75 with angiographically proven CAD (≥70% lesion in at least one major coronary artery or major branch), plasma T concentration ≤12 nM (346 ng/dL); 2 dropouts prior to medication tx were not analyzed. One subject had unanalyzable data.	160 mg/day T undecanoate or placebo by mouth for 8 weeks followed by tx switch. Randomization method not given.	No change in plasminogen activator-1, fibrinogen, or factor VII.	4
**Guler et al, 2006**[28]	41 men with CAD who underwent stenting.	3 weekly IM doses of T (n = 25; Sustanon 250 = T propionate 30 mg, phenylproprionate 60 mg, isocaproate 60 mg, and decanoate 100 mg); 3-week interval before stenting with usual tx (n = 16). Described as double-blind, but no placebo injection discussed. Randomization method not given.	24 hours after stenting,↓interleukin-6 (IL-6), ↓C-reactive protein. No effect on TNF-α.	2
**Nakhai-Pour et al, 2007**[29]	237 men age 60–80 with serum T concentration below the population median (13.7 nM); 14 were lost to follow-up.	160 mg daily T undecanoate 160 mg/day (n = 113) or placebo (n = 110). Randomization methods not given.	No effect of tx on C-reactive protein, except ↑ in men with baseline C-reactive protein concentration below the median.	5
**Frederiksen et al, 2012**[30]	38 men age 60–78 with free T concentration <7.3 nM finished the study. Number starting not given.	Unspecified dose of T gel or placebo used for 6 months.	↓Osteoprotegerin. No change in C-reactive protein.	3
**Gianatti et al, 2014**[32]	88 men age 35–70 years of age with a history of T2DM and total testosterone ≤12.0 nML (346 ng/dL). 13 men did not complete the study, 8 because of intensification of oral hypoglycemic agents or commencement of insulin therapy. 1 subject in testosterone group was withdrawn with a hematocrit of >54 prior to his 30 week injection.	Participants were randomly assigned in a concealed 1:1 allocation to T or placebo using permuted blocks with a block size of 4. IM T undecanoate 1000 mg (n = 45) or placebo (n = 43) at 0, 6, 18, and 30 weeks.	No change in CRP concentration.	4

#### 3.1.1 Coronary artery disease

In studies that investigated the effect of testosterone on patients with coronary artery disease (CAD), eligible men generally were identified based on stable angina, angiographic evidence of some degree of coronary artery occlusion, or a history of myocardial infarction (MI). Six studies involved men in whom the study authors reported evidence of hypogonadism either clinically [1] or based on plasma testosterone concentration [2–6]; the remainder included men without regard to plasma testosterone concentration. All but three of the studies evaluated ST-segment depression on an exercise stress test using a modification of the Bruce protocol. One of the studies not using the Bruce protocol evaluated findings on electrocardiography (ECG) and Holter monitoring, without specification of an exercise protocol.[7] Another study added single-photon emission computer tomography (SPECT) to evaluate for deficits in myocardial uptake of a labeled perfusion tracer.[8] One study used magnetic resonance imaging (MRI) estimates of myocardial perfusion.[3] One study evaluated change in coronary artery calcium score over time, showing no difference between testosterone and placebo.[6]

Two studies evaluated brachial artery response to release of occlusion as an indicator of sensitivity to local vasodilators in men with CAD and did not directly address the coronary arteries; both reported results favorable to testosterone therapy.[9, 10] Two studies in apparently healthy men with bioavailable testosterone <4.44 nM (128 ng/dL) or total testosterone ≤15 nM (432 ng/dL) found no change in brachial artery reactivity in response to transdermal testosterone or dihydrotestosterone therapy.[5, 11] The study that used MRI showed no effect of 8 weeks of oral testosterone undecanoate therapy on myocardial perfusion, although there was increased perfusion of those segments supplied by an unobstructed coronary artery.[3]

Three studies used acute treatments with intravenous (IV) testosterone just prior to exercise testing. Two of the studies showed favorable effects of treatment on time to ST-segment depression.[2, 12] One study showed no effect on ECG or SPECT evidence of ischemia.[8] A year-long study showed benefits of testosterone treatment on ST-segment depression.[4] The remaining eight studies evaluated treatments of 2 to 24 weeks in duration.[1, 3, 10, 11, 13–17] The 3 studies that looked at time to ST-segment depression found a benefit of testosterone supplementation.[1, 14, 16]

Although 2 studies reported improvements in angina symptoms during or after testosterone treatment,[7, 15] 4 studies showed no effect of treatment on angina.[1, 4, 8, 16] Most studies did not report any measure of angina symptoms. A study on men with leg claudication or trophic ulcers attributed to arteriosclerosis did not show an improvement in subjective symptoms, walking, or plethysmographic estimation of blood flow endpoints after 3 months of testosterone therapy.[13]

There was a decreased incidence of silent MI with testosterone treatment in 1 study.[15] Another study, designed to determine the effect of testosterone supplementation on lower-extremity strength and physical function in men 65 years of age and older, was stopped early by a Data and Safety Monitoring Board due to an excess of cardiovascular adverse events.[17] These adverse events included acute coronary syndrome (ACS), MI, ECG abnormalities, and arrhythmias, among others.

Eleven studies of coronary artery disease scored 4 or 5 on the Jadad scale. Of these, only one of five studies that included angina as an outcome found a benefit. Four of five studies that assessed ST segment depression found a benefit.

#### 3.1.2 Congestive heart failure

Six studies evaluated effects of testosterone treatment on CHF.[18–23] In two papers from the same group,[19, 20] it is not clear whether treatments were randomly assigned. Administration of testosterone by the buccal route was associated with beneficial effects on cardiac index and systemic vascular index in the acute catheterization setting, consistent with an acute vasodilatory effect.[19] Intramuscular (IM) testosterone treatment for 12 weeks improved exercise capacity and reduced heart failure symptom scores without identifiable effects on left ventricular size or ejection fraction (EF).[20] Another study of IM testosterone in men with CHF showed an improvement in oxygen consumption, respiratory efficiency (ventilation/carbon dioxide consumption), and distance walked in 10 minutes without changes in EF or left ventricular end-diastolic diameter.[18] The improvements in exercise function appeared attributable to the response of men with baseline plasma testosterone concentration <12 ng/mL (~4 nM). A study of a testosterone patch showed improvement in the shuttle walk test.[23] Another study showed no effect of IM testosterone enanthate on ejection fraction, although there was an improvement in a Doppler-based myocardial performance index.[22]

The only study that scored above a 3 on Jadad found a benefit on CHF measures.[18]

#### 3.1.3 Lipids

Serum or plasma concentrations of cholesterol fractions, triglycerides, and lipoproteins have been used as surrogate endpoints for cardiovascular risk, although they should not be mistaken for markers of cardiovascular adverse events. In 25 studies, testosterone treatment was associated with favorable, unfavorable, or no effects on lipids as summarized in Table 1. Favorable effects in 11 studies included 5–11% decreases in total cholesterol concentration and variable and inconsistent decreases in triglycerides and low-density lipoprotein (LDL) cholesterol. One of the studies counted as showing a favorable effect did not demonstrate a change in total or high-density lipoprotein (HDL) cholesterol or triglycerides but reported a 13% reduction in LDL cholesterol.[24] This finding was based on averages of several repeated measurements over the course of 1 year rather than a determination of improved lipid measurements at the end of the treatment period.

Unfavorable changes were reported in 2 studies [5, 25] and included increases in total cholesterol, LDL cholesterol, and triglycerides and decreases in HDL cholesterol in men evaluated as hypogonadal prior to androgen therapy. Most of the studies that did not report favorable effects of testosterone on lipids reported no effects at all. One of these studies[26] reported a decrease in lipoprotein-a (LP-a), but this finding was transient and occurred in a study with multiple measurements at multiple time points in multiple patient subgroups without adjustment for multiple comparisons. Nine of the 11 studies that had favorable effects on lipids had Jadad scores of 4 or 5. Nine of the 14 studies that lacked favorable effects on lipids had Jadad scores of 4 or 5.

The discordance between studies on the lipid effects of testosterone treatment did not appear to be route dependent. Seven of the 11 studies showing favorable effects used IM injection of testosterone enanthate, esters, or undecanoate. Five of the 14 studies not showing favorable effects on lipids used IM injection of testosterone esters, cypionate, or undecanoate.

#### 3.1.4 Inflammatory or coagulation markers

Eleven studies were identified in which markers that have been associated with atherosclerotic cardiovascular disease risk were measured in men using testosterone or dihydrotestosterone therapy. Three studies reported favorable effects of testosterone on tumor necrosis factor-α (TNF-α), a marker of inflammation.[1, 3, 27] One study in men with CHF showed no effect of testosterone treatment by buccal, IM, or transdermal routes on serum concentration of TNF-α.[21] One of the studies asserted that there was a decrease in the inflammatory marker interleukin-1β (IL-1β), but a statistically significant effect was not shown.[1] Another study showed a decrease in interleukin-6 (IL-6) and C-reactive protein, additional inflammatory markers.[28] Two studies performed in elderly men who were largely without a diagnosis of CAD showed no beneficial effect of testosterone therapy on C-reactive protein[29, 30] as did two studies of men with type 2 diabetes mellitus.[31, 32] Transdermal dihydrotestosterone did not affect inflammatory markers in men with low total pretreatment testosterone concentrations.[33] No change in fibrinogen, plasminogen activator inhibitor-1, or tissue plasminogen activator was shown in men with CAD who used testosterone patches or oral doses.[3, 34]

### 3.2 Sexual Function

The 48 studies that assessed sexual function or libido as a primary or secondary endpoint are summarized in Table 2. Study populations included men identified by study authors as “hypogonadal,” normal men, and men with erectile dysfunction (ED). Studies included men with depression,[35–37] chronic renal disease,[38] cirrhosis,[39] arterial insufficiency,[40] cancer,[24] diabetes,[26] HIV,[35, 41] Alzheimer disease,[42] and chronic obstructive pulmonary disease (COPD).[43] Preparations included IM (n = 16), oral (n = 11), topical gel or solution (n = 14), patch (n = 5), and buccal (n = 1) formulations. Studies used a variety of questionnaires, including the International Index of Erectile Function (IIEF), Frenken sexual experience scales, Derogatis Sexual Performance Scale (DSPS), the Aging Males’ Symptoms (AMS) scale, Male Sexual Health Questionnaire, Psychosexual Daily Questionnaire, and study-specific questionnaires. Study reports used different language for symptoms, so we grouped, for example, “libido,” “sexual interest,” and “sexual desire.”

**Table 2:** Effects of Testosterone on Sexual Functioning.

**Hentzer & Madsen, 1967**[40]	39 males with arterial insufficiency; 3 dropouts.	200 mg T (n = 19) or placebo (n = 17) IM weekly for 3 weeks, then once every second week for 6 months; consecutively enrolled patients assigned alternately to T or placebo by record numbers.	Improved sexual function in 9 T-treated (n = 19) and 2 placebo-treated (n = 17) men (Fisher *P* = 0.03 calculated by us, not by authors).	3
**Benkert et al, 1979**[62]	36 men 45–75 (mean age 56.5) with erectile dysfunction (ED); 7 men withdrew, 2 in the placebo group and 5 in the T group.	120 mg/day T undecanoate (80 mg qam and 40 mg qpm; n = 18) or placebo (n = 18) PO for 8 weeks; 2-week placebo run-in; 2-week placebo withdrawal phase, and 8 additional weeks of observation.	No difference from placebo in ability to have an erection or sexual satisfaction (both groups improved).	3
**Davidson et al, 1979**[44]	6 hypogonadal men with T <150 ng/dL (5.2 nM).	Crossover, within-subject, 3 phase design. T enanthate 0, 100, or 400 mg IM each administered once, a month apart. Tx were administered in “arbitrarily chosen order and “varied at random within and among subjects.”	400 mg dose improved total erections, nocturnal erections, and coital frequency. No effect on incidence of orgasm or masturbation.	3
**Skakkebaek et al, 1981**[45]	12 men 22–48 years old diagnosed as androgen deficient; one man subsequently found to be normal and excluded.	160 mg T undecanoate or placebo daily by mouth for 2 months followed by tx switch; randomization of order of tx not described but said to be balanced.	↑Sexual acts and ejaculations per week. ↑Frequency of sexual thoughts and excitement. No effect on subjective quality of sexual act. Subjective ratings of sexual enjoyment, erectile and ejaculatory problems improved.	3
**Nankin et al, 1986**[46]	10 men 51–74 years old with secondary impotence and T concentrations <420 ng/dL (14.6 nM).	200 mg T cypionate or placebo IM every 2 weeks for 12 weeks followed by the opposite tx; no washout period mentioned, randomization of tx order not described.	↑Libido and potency based in part on scores assigned to questionnaire responses analyzed using t-test; insufficient detail provided to permit nonparametric analysis of ranked data.	4
**Gluud et al, 1988**[39]	221 alcoholic men 24–79 years old (median age 53) with cirrhosis; dropouts not described but range of follow-up was 1–48 months; results for 110 men were included at 30 months.	600 mg T (not otherwise specified) in divided doses or placebo by mouth daily for a median of 30 months; randomization 3:2 (T:placebo); no other details about number in each group or randomization method.	No effect on libido, erectile or ejaculatory function.	3
**Anderson et al, 1992**[63]	31 healthy men, 21–41 years old in a male contraceptive study.	200 mg T enanthate (n = 16) or placebo (n = 15) IM weekly for 4 weeks (n = 16); continuation of study with T in place of placebo not discussed here; randomization method not given.	↑Sexual interest on the psychosocial stimulation scale of the 4-part Frenken Sexual Experience Scales (FSES). No effect on the other 3 parts of the FSES. No effect on masturbation, intercourse or waking erection.	2
**Holmäng et al, 1993**[79]	25 obese men 40–65 years old, mean age 52years; 2 dropped out, 1 in each group.	320 mg T undecanoate or placebo daily for 8 months; randomization procedure not described.	Five in the T group (n = 11) vs 1 in the placebo group (n = 12), “reported a subjective feeling of increased muscular energy and sexual desire.” No other details provided. (Fischer’s exact test not significant when performed by us; not analyzed by authors.)	4
**Aydin et al, 1996**[64]	79 men (mean age 34.2–39.5) with non-organic ED; unclear how many withdrew.	120 mg/day T undecanoate (n = 20) or placebo (n = 18) daily presumably by mouth (trazodone and hypnosis arms not discussed here); 12- and 16-week results not represented due to high dropout rate; study analyzed only at 4 and 8 weeks; randomization method not given.	At 8 weeks, 8 in the T group (n = 20) and 6 in the placebo group (n = 18) were considered “cured;” 4 T vs 1 placebo experienced a moderate response; and 8 T vs 11 placebo had no response (χ² not significant by us; not analyzed by authors).	2
**Schiavi et al, 1997**[59]	18 men 46–67 years old (median age 60 years) with ED with or without hypoactive sexual desire; 12 men completed the study, dropouts were excluded from analysis.	200 mg T enanthate or placebo IM every 2 weeks for 6 weeks followed by 4-week washout followed by the opposite tx; order was randomized by unstated method; 7 subjects received placebo first, 5 subjects received T first.	No effect on sexual satisfaction, frequency of sexual desire, masturbation, sex with partner, morning erections, or degree of erections. ↑Ejaculations.	3
**Dobs et al, 1998**[65]	13 men with T concentrations <250 ng/dL (8.7 nM) who had previously received IM T therapy; 1 man dropped out after 4 weeks.	10 mg T or taste-matched placebo (pseudoephedrine 3 mg) buccal tablet daily for 4 weeks; doubling of dose permitted after end of first 4 weeks; randomization method not given.	No effect on total scores of Watts Sexual Function Questionnaire (WSFQ) after 8 weeks; ↑Frequency of sexual desire and morning erections, but not erections in general; ↑Maximum rigidity and duration of full nocturnal penile tumescence assessed in a sleep lab.	3
**Rabkin et al, 2000**[35]	74 HIV-positive men, mean age 39, with serum T <17.4 nM (501 ng/dL) with sexual dysfunction and at least one “hypogonadal” mood symptom; 3 subjects in the placebo group dropped out, 1 in T group was excluded from analysis because of medication error. Among the 70 subjects analyzed, 26 had major depressive disorder (MDD), dysthymia, “minor” depression, or MDD in remission.	Randomization in blocks of 4 by computer-generated numbers to T cypionate (n = 38 overall, 26 with depression diagnosis) or placebo (n = 32 overall, 7 with a depression diagnosis) injected biweekly (presumably IM) for 6 weeks; the first T dose was 200 mg, subsequent doses were 400 mg. Open-label phase with T followed the 6-week double-blind study and is not summarized here.	↑Libido and morning erections. Testosterone improved ED by expanded CGI (Clinical Global Index) among completers with initial ED.	4
**Seidman et al, 2001**[36]* Seidman & Roose, 2006[164]	32 men 33–71 years old with MDD (DSM-IV criteria) and serum T ≤350 ng/dL (12.1 nM); 2 dropouts in the T group and 1 in the placebo group were excluded from analysis.	200 mg T enanthate (n = 13) or placebo (n = 17) IM weekly for 6 weeks. Randomization method not mentioned.	No effect on Derogatis Sexual Performance Scale (DSPS).	4
**Howell et al, 2001**[24]	35 men mean age 40.9 years with some degree of testicular dysfunction after cytotoxic cancer therapy; blood LH concentration ≥8 mIU/L and T concentration <20 nM (576 ng/dL); 2 dropouts, 1 in each group, were excluded from analysis.	2.5 or 5.0 mg T patch (n = 16) or placebo patch (n = 19) daily for 12 months; randomization method not described, study described as single-blind.	No effect on interest in sex, sexual activity, or frequency of erections.	3
**Park et al, 2003**[66]	39 men with sexual dysfunction, infertility, symptoms of hypogonadism and T <400 ng/dL (13.9 nM); 4 subjects in the T group dropped out.	160 mg T undecanoate (n = 33) or placebo (n = 6) daily by mouth for 3 months; described as single-blind, randomization method not given.	Between-group analyses not reported. Fischer’s exact test as performed by us showed no significant difference between groups in showing sexual function improvement. Androgen Deficiency in the Aging Male (ADAM) questionnaire scores reported improvement only in the treated group, but total scores not provided.	2
**Aversa et al, 2003**[47]	20 men 48–66 years old (mean 56 years old) with arteriogenic ED and T 10–13 nM (288–375 ng/dL) and free T 200–300 pM (5.8–8.6 ng/dL) who had not responded to sildenafil.	5 mg transdermal T (n = 10) or placebo (n = 10) patch daily for 1 month; all subjects received sildenafil 100 mg to use on demand; randomization method not given.	Improved overall erectile function domain score, intercourse satisfaction, overall satisfaction, number of acts of sexual intercourse, and percentage of successful intercourse attempts. Improved erections in 8 men on T (n = 10) and 1 on placebo (n = 8). No change in sexual desire and orgasmic function.	3
**Steidle et al, 2003**[56]	406 hypogonadal men 20–80 years old, mean age 60.5 years in T group, 56.8 years in placebo group, with T concentration ≤10.4 nM (300 ng/dL) and at least one symptom of fatigue, decreased muscle mass, reduced libido, or reduced sexual functioning; 6 patients in the T gel group discontinued because of AEs.	50–100 mg T (n = 99; 43 titrated up to 100 mg) or 100 mg (n = 106; 4 titrated down to 50 mg) or placebo (n = 99) gel daily for 90 days; dose titration took place at 60 days; randomization method not given; open-label portion of study not discussed here.	100 mg group, but not 50 mg group, was superior to placebo for spontaneous erections, sexual motivation, desire, and performance at 90 days. No change in sexual enjoyment with or without a partner, satisfaction with erection duration, or percentage of full erection.	4
**Cavallini et al, 2004**[48]	150 men >50 years old with symptoms consistent with hypogonadism and free T <6 pg/mL (21 pM); 20 dropouts analyzed by ITT.	160 mg/day T undecanoate (n = 40), propionyl-L-carnitine 2 g/day plus acetyl-L-carnitine 2 g/day (n = 45), or placebo (n = 45) presumably by mouth for 6 months; randomization method not given.	↑Erectile function at 3 and 6 months, sexual desire at 3 but not 6 months, and sexual intercourse satisfaction at 6 but not 3 months. No change in general sexual well-being or orgasm. Carnitine superior to T for erectile function at 3 and 6 months and for orgasm and general sexual well-being at 6 but not 3 months.	4
**O’Connor et al, 2004**[67]	28 healthy eugonadal men 22–44 years old; 4 subjects treated with T withdrew and were excluded.	1000 mg T undecanoate or placebo IM at the beginning of an 8-week phase, followed by 8-week washout, followed by the opposite tx, randomization of tx order not described.	No effect on frequency of sexual intercourse, masturbation, sexual desire, enjoyment of intercourse, or overall satisfaction with sexual experience on weekly logs.	3
**Shabsigh et al 2004**[69]	75 hypogonadal men 41–66 years old with T ≤400 ng/dL (13.9 nM) with ED who had failed to respond to sildenafil; 12 subjects withdrew, 5 in placebo group, 7 in T group.	1% T (n = 39) or placebo (n = 36) gel as adjunctive therapy to sildenafil 100 mg for 12 weeks, randomization by computer generated schedule stratified by researcher.	No effect on mean change from baseline in total International Index of Erectile Function (IIEF) scores and erectile function, orgasmic function, and overall satisfaction domains at 8 weeks or 12 weeks. No differences between groups in average number of successful sexual attempts or self-assessed improvement in erection.	5
**Svartberg et al, 2004**[43]	29 men, mean age 67.5 years in the placebo group, 64.5 years in the T group with moderate to severe COPD; 2 dropped out, 1 in each group.	250 mg T (n = 15) or placebo (n = 14) IM every 4 weeks for 26 weeks; method of randomization not discussed.	Improved score on IIEF and improved erectile function. ↑Sexual quality of life at 12 but not 26 weeks.	4
**Haren et al, 2005**[69]	76 men 60–86 years old, mean age 68.5 years with ≥ 2 “androgen deficiency” symptoms, total T >8 nM (231 ng/dL) and free T index between 0.3 and 0.5.	80 mg T undecanoate or placebo PO twice daily for 12 months, number in each group unclear, randomization with a block of 4 was used “in the Almedica drug labeling system (ADLS).”	No effect on symptom change from baseline.	4
**Brockenbrough et al, 2006**[38]	40 men with anemia of chronic renal disease (mean age 59 years in T group and 53 years in placebo group) on erythropoietin with serum T <300 ng/dL (10.4 nM); 10 withdrew in the placebo group and 8 withdrew in the T group.	T 100 mg (n = 19) or placebo (n = 21) gel daily for 6 months; block randomization in groups of four stratified by erythropoietin dose at 250 U/kg/week; details of randomization not given.	No effect on sexual function, desire, relationships, or behavior.	5
**Lu et al, 2006**[42]	47 men (18 with Alzheimer disease (AD) and 29 healthy men); mean age 69.3 years (T) and 70.3 years (placebo) in those with AD; 63.6 years (T) and 61.2 years (placebo) in healthy men; 6/23 in T group and 3/25 in placebo group withdrew.	75 mg T gel (n = 9 with AD and 14 healthy men) or placebo (n = 9 with AD and 15 healthy men) gel daily for 24 weeks; method of randomization not given.	No effect on sexual functioning, by the modified Change in Sexual Functioning Questionnaire (CSFQ). Caretakers of AD patients reported no sexual behavior changes.	3
**Merza et al, 2006**[49]	39 men 44–77.4 years old, mean age 62 years, with sexual dysfunction and total T <10 nM (288 ng/dL) or free androgen index <30%; 8 patients withdrew, 4 in each group.	T patch 5 g/day (n = 20) or placebo patch (n = 19) for 6 months; randomization list “generated by Biostatistical Department at Ferring AB.”	Significant difference between groups in Male Erectile Dysfunction Quality of Life questionnaire (MEDQoL) due to decrement in placebo group (no change from baseline in patch group).	3
**Chiang et al, 2007**[50]	40 men 20–74 years old with serum T <300 ng/dL (10.4 nM) or free T <8.7 pg/mL (30 pM); 4 subjects withdrew, all in placebo group.	T 50 mg (n = 20) or placebo (n = 20) gel daily for 3 months; randomization method not given.	↑Sexual function on comparison made to baseline. No effect on magnitude of score increase with t-test performed by us.	3
**Chung et al, 2007**[61]	30 healthy men 18–45 years old.	T 200 mg mixed esters (n = 10) or placebo (n = 10) IM weekly for 4 weeks; nandrolone arm not discussed here; computer-generated randomization in blocks of 6.	No effect on sexual function.	5
**Allan et al, 2008**[70]	62 healthy men ≥55 (mean age 62.1 years in the placebo group and 64.5 years in placebo group) with serum T <15 nM (432 ng/dL); 14 in the treated group and 6 in placebo group dropped out.	5 mg transdermal T (n = 31) or placebo (n = 31) patch daily for 52 weeks. Subjects randomized in a 1:1 ratio; randomization method not given.	↑Sexual desire at 26 and 52 weeks. No effect on any erectile function, orgasmic function, intercourse satisfaction, overall satisfaction.	5
**Knapp et al, 2008**[41]	61 HIV-positive men, mean age 43 years, with involuntary weight loss and/or body-mass index <20 mg/m^2^; 4 placebo-assigned and 9 T-assigned subjects dropped out and were analyzed using the last observation carried forward.	300 mg T enanthate (n = 31) or placebo (n = 30) weekly for 16 weeks; randomized by computer-generated list in blocks of 6.	No difference between groups in sexual function assessed by a 5-question scale: 1) “I found it easy to achieve an erection when I wanted to”; 2) “I have lost interest in sex”; 3) “I found it difficult to sustain an erection when I wanted to”; 4) “I had problems achieving an orgasm”; and 5) “I am generally satisfied with the sex that I have.”	5
**Emmelot-Vonk et al, 2009**[71]	237 healthy men, 60–80 years old (mean 67) with T <13.7 nM (395 ng/dL); 30 dropped out; 16 in T group, 14 in placebo group.	160 mg T undecanoate (n = 120) or placebo (n = 117) in divided daily oral doses for 26 weeks; randomization computer-generated using blocks of 6 (Emmelot-Vonk et al[91]).	No effect on sexual functioning including frequency of sexual activity, quality of sexual functioning, and ability to achieve or maintain erection.	5
**Legros et al, 2009**[57]	322 men ≥50 years old with symptoms of hypogonadism and calculated free T <0.26 nM (7.5 ng/dL); 243 completed, 310 analyzed for efficacy, 39 discontinued due to AEs and 22 withdrew consent; no marked difference between groups in discontinuation rates.	PO T undecanoate 0 (n = 79), 80 (n = 78), 160 (n = 82), or 240 mg (n = 77) daily for 12 months; randomized in unstratified blocks.	Improved sexual symptoms subscale score of Aging Males Symptoms (AMS) scale only in middle dose group (160 mg/day).	4
**Morales et al, 2009**[72]	86 men 45–70 years old with sexual dysfunction and T <12 nM (346 ng/dL); 7 withdrew; 5 in T group, one each in DHEA and placebo groups.	160 mg T undecanoate (n = 29) or placebo (n = 29) daily by mouth for 4 months; DHEA arm not further discussed here; randomization in permuted blocks of 9.	No effect on IIEF, ADAM, AMS, or Global Assessment Questionnaire (GAQ).	5
**Seidman et al, 2009**[51]	23 men, mean age 50.6 years, with dysthymia and serum T concentration <350 ng/dL (12.1 nM).	T cypionate 200 mg (n = 13) or placebo (n = 10) IM every 10 days for 6 weeks; randomization by computer.	A significant time by tx interaction in the IIEF total score demonstrable in all sexual function domains, but no data provided.	5
**Buvat et al, 2011**[55]	223 men with ED and total T ≤4 ng/mL (13.9 nM) or bioavailable T ≤1 ng/mL (3.5 nM) who had not responded adequately to highest dose of a phosphodiesterase inhibitor; 35 withdrawals (20 placebo, 15 T).	After 4-week valdenafil run-in, patients randomized to T 50 mg (n = 83) or placebo (n = 84) gel daily for 12 weeks; dose doubled to 100 mg at 4 or 8 weeks based on clinical response; randomization prepared by manufacturer, method not given.	No improvement in erectile function by intention-to-treat (ITT) analysis (both groups improved). ↑Intercourse satisfaction in men with baseline T <3 ng/mL (10.4 nM) in *post hoc* analysis.	3
**Giltay et al, 2010**[52]	184 men 35–69 years old (mean 52.1 years old) with T concentration <12.0 nM (346 ng/dL) or calculated free T concentration <225 pM (6.5 ng/dL) who had metabolic syndrome; 14 dropouts (8 T, 6 placebo) were analyzed using last observation carried forward.	T undecanoate 1000 mg (n = 113) or placebo (n = 71) IM given every week 0, 6, 18, and 24; evaluations at week 30. Randomization method not given except to say that T was over-assigned in a 7:3 ratio.	↑AMS scale and IIEF	4
**Srinivas-Shankar et al, 2010**[53]	274 frail men, mean age 74 years, with total T ≤12 nM (346 ng/dL) or free T ≤250 pM (7.2 ng/dL); 31 withdrawals, 15 placebo, 16 T.	50 mg T (n = 138) or placebo (n = 136) gel daily for 6 months; day 10 and 3 months adjusted to 25–75 mg based on T; computerized randomization in blocks of 10.	↑AMS sexual domain based on adjusted differences between groups; total AMS scores not provided.	5
**Amiaz et al, 2011**[37]	100 depressed men age 30–65 (mean 50.7 in placebo group, 51.5 in treated group) treated with serotonergic antidepressants, with serum T <350 ng/dL (12.1 nM); data lost (n = 26); 5 withdrew, and 6 apparently had incomplete records.	T gel (amount and dose not described; n = 50) or placebo gel (n = 50) for 6 weeks; randomization by table of random numbers with a blocked design of 50.	↑Erectile function, intercourse satisfaction, orgasmic function, sexual desire, overall satisfaction, total IIEF.	5
**Ho et al, 2011**[73]	120 Malaysian men age 40–70 (mean 53) with "mild" symptoms on AMS scale and total T <12 nM (346 ng/dL); 8 dropouts, 4 per group.	1000 mg T undecanoate (n = 56) or placebo (n = 58) IM at week 0, 6, 18, 30, and 42; randomization method not described	No effect on the AMS sexual domain scores. Total scores improved.	4
**Jones et al, 2011**[26]	220 men, mean age 59.9, with metabolic syndrome or T2DM or both and total T ≤11 nM (317 ng/dL) or free T <255 pM (7.3 ng/dL); 54% of subjects completed the study, ITT analysis used last observation carried forward.	60 mg T (n = 108) or placebo (n = 112) gel for 12 months; randomization stratified by presence of metabolic syndrome only, DM only, and DM with metabolic syndrome; doses adjusted based on T measurements.	Improved sexual desire, intercourse satisfaction, and total IIEF scores. No effect on AMS, erectile or orgasmic function, or overall sexual satisfaction scores.	4
**Spitzer et al, 2012**[74]	140 men 40–70 years old, mean age 55.1 years for T group, 54.6 years for placebo group, with low erectile function scores and total T concentrations <11.45 nM (330 ng/dL) or free T concentrations <173.35 pM (5 ng/dL); 22 dropped out, 10 T and 12 placebo.	5–15 g T 1% gel (n = 70) or placebo (n = 70) gel daily for 14 weeks; randomization sequence used blocks of 4; other details not provided; all subjects received sildenafil.	No effect of T on sexual function, assessed by questionnaires, frequency of sexual encounters, percentage of successful sexual intercourse, and other measures.	5
**Del Fabbro et al, 2013**[75]	43 men with advanced cancer and bioavailable T <70 ng/dL (2.4 nM), hemoglobin >9 g/dL, Eastern Cooperative Oncology Group performance status <3, and moderate to severe fatigue; 3 placebo and 5 T (1 died) dropped out.	T enanthate 150 or 200 mg (depending on weight; n = 19) or placebo (n = 24) IM every 2 weeks for 2 months. Doses titrated to a bioavailable T goal of 70–270 ng/dL (2.4–9.4 nM).	No change on Sexual Desire Inventory (SDI) (*P* = 0.054).	4
**Hackett et al, 2013**[54]	199 men, mean age 61.6 years, with total T 8.1–12 nM (233–346 ng/dL) or free T 0.181–0.25 nM (5.2–7.2 ng/dL) or total T ≤8.0 nM (231 ng/dL) or free T ≤0.18 nM (5.2 ng/dL); 9 dropouts, 4 in the active group and 5 in placebo group.	T undecanoate 1000 mg (n = 97) or placebo (n = 102) IM at weeks 0, 6, and 18; evaluated at 18 & 30 weeks; randomized in unstratified blocks.	At 30 weeks, ↑erectile function, intercourse satisfaction, sexual desire, orgasm; no effect on overall satisfaction; no changes in AMS.	5
			At 18 weeks, ↑intercourse satisfaction, sexual desire, and orgasm; no effect on ED, overall satisfaction; or AMS.	
**Basaria et al, 2015**[6]	Men aged 60 years or older, morning total T 100–400 ng/dL (3.5–14 nM) or free T <50 pg/mL (1.7 pM). 1:1 concealed randomization with stratification by age dichotomized at 75 years and by site. Computer-generated randomization. All subjects receiving at least 1 medication dose were retained for analysis.	3 years of daily application of 75 (n = 155) or 0 (n = 151) mg testosterone as a gel, dose level adjusted upwards or downwards based on total testosterone 2–12 hours after gel application. Placebo adjusted by an unblinded observer. 44/155 receiving T did not complete, 23 for adverse events; 51/151 receiving placebo did not complete, 17 for adverse events.	No difference in IIEF total score or subscales except intercourse satisfaction increased more with T than with placebo	5
			No difference in marital interaction scale from the Cancer Rehabilitation Evaluation System Short Form	
**Paduch et al, 2015**[78]	Sexually active men 26 or more years old with ejaculatory dysfunction and total T< 300 ng/dL (10.41 mM).	T solution applied to axilla daily at 60 (n = 39) or 0 (n = 35) mg/day, titrated up or down based on serum T concentration after 4 weeks. Computer randomization scheme on a 1:1 basis. Five subjects in each group discontinued, 1 in T group due to adverse event.	No difference in changes in Male Sexual Health Questionnaire-Ejaculatory Dysfunction-Short Form or individual components of the total score; No change in ejaculate volume; No differences in ejaculation or orgasm components of the IIEF; No differences in sexual activity log	5
**Snyder et al, 2016**[60]	790 men ≥65 with a serum total T concentration of <275 ng/dL and symptoms suggesting hypoandrogenism. 705 completed.	T gel (1%) or placebo x 1 year. Initial dose was 5 g daily but dose was adjusted to keep T concentration within range for young men. Dose was changed simultaneously in subject taking placebo. Number of subjects in each arm cannot be determined.	↑ sexual activity; ↑ sexual desire and erectile function; No benefit on vitality assessed by Functional Assessment of Chronic Illness Therapy-Fatigue scale; No benefit on physical function.	2
**Basaria et al, 2015**[77]	84 men, 18–64 using opioid analgesics for chronic noncancer pain, with a morning serum total T concentration of <350 ng/dL (12 nM)	T gel (1%) 5 (n = 43) or 0 (n = 41) g daily x 3 months. In T group, dose adjusted at two weeks by unblinded physician if T <500 ng/dL. Unblinded physician then increased dose of a participant in the placebo group. Study completion by 36 on T and 29 on placebo.	↑Desire; No effect on erectile function or orgasmic domain of IIEF.	4
**Gianatti et al, 2014**[76]	88 men with T2DM, age 35–70, with total testosterone <346 ng/dL (12 nM), aging male symptoms, and erectile dysfunction; 75 completed.	T undecanoate 1000 (n = 44) or 0 (n = 41) mg IM at week 0, 6, 18, and 30, randomized using permuted blocks.	No improvement in Aging Males Symptoms total score, sexual subscore, or sexual desire. Restriction of analysis to symptomatic men revealed no difference. Erectile function was reduced compared with placebo, but no change compared to baseline.	4
**Hackett et al, 2014**[58]	199 men aged 18–80 with T2DM with total T between 8.1–12 nM (233–346 ng/dL) or total T of ≤8.0 nM (231 ng/mL). 9 patients did not complete the study; 4 because of serious adverse events (3 treatment unrelated deaths, 1 prostate cancer in placebo), and 5 withdrew their consent.	Subjects were block randomized to receive testosterone undecanoate 1000 (n = 97) or 0 (n = 102) mg IM at week 0, 6, and 18.	Improvement in erectile function, intercourse satisfaction, sexual desire, and AMS in group starting with total T ≤8.0 nM; Improved AMS score but no improvement in sexual function in group starting with total T between 8.1 and 12 nM.	5

Of 47 studies that assessed sexual function or satisfaction, 23 studies reported beneficial effects of testosterone treatment for at least 1 measure of sexual function or satisfaction,[6, 26, 35, 37, 40, 43–60] and 24 studies did not show testosterone-associated improvements in any sexual function endpoint.[24, 36, 38, 39, 41, 42, 61–78] Three studies we counted as positive were mixed: Steidle et al found improvement with 100 but not 40 mg of gel, Legros et al[57] tested 3 dose levels of orally administered testosterone undecanoate (60 mg, 160 mg, and 240 mg) and found a benefit only for the middle dose, and Hackett et al[58] found that testosterone worked in a group with testosterone ≤8.0 nM for intercourse satisfaction but not in a group with testosterone 8.1–12 nM. One study “reported a subjective feeling of increased muscular energy and sexual desire in some subjects”.[79] There was no difference between groups by Fischer exact test (performed by us) and we excluded this study from further analysis. Limiting analysis to the 30 studies with Jadad scores of 4 or 5 yielded similar results; 14 were positive and 16 negative.

Of 31 studies that evaluated erectile function, 15 found no improvement with testosterone therapy,[6, 26, 39, 41, 55, 59, 62, 64, 65, 68, 70–72, 76, 77] and 16 reported a benefit.[35, 37, 43–52, 56, 58, 60] Although the study by Chiang et al[50] reported a benefit of both testosterone and placebo compared to baseline; however, our analysis did not show a difference between treatment groups. Limiting analysis to the 17 studies with Jadad scores of 4 or 5 yielded similar results; 9 were positive and 8 were negative.

Twelve studies included men with ED; 8 found no benefit of testosterone over placebo,[55, 59, 62, 64, 68, 72, 74, 76] and 4 found a benefit.[35, 46, 47, 49] One negative study found that testosterone reduced erectile function when compared to placebo; however, there was no change when each group was compared to its baseline.[76]

Of 23 studies that specifically reported changes in libido, 13 found that testosterone treatment increased libido,[26, 35, 37, 45, 46, 54, 56, 60, 63, 65, 70, 77, 79] eight found no effect,[24, 38, 39, 47, 59, 67, 75, 76] and 1 found an effect after 3 but not 6 months of treatment.[48] Hackett et al[58] found that testosterone improved sexual desire in a group with initial testosterone ≤8.0 nM but not in a group with initial testosterone 8.1–12 nM.

Eleven studies used the Aging Males’ Symptoms scale, which includes 3 questions on libido and sexual function. Five studies found no difference between testosterone and placebo on total scores,[26, 54, 57, 72, 76] and 4 studies found a benefit of testosterone.[52, 57, 58, 66] One paper[53] reported only sexual subscales but not total AMS scores. On the sexual subscale of the AMS scale, this study reported a benefit, Ho et al[73] found no benefit, and Legros et al[57] found a benefit of testosterone on the AMS sexual subscale only in the middle (160 mg) of 3 dose levels at 3 of 4 time points. Hackett et al[58] found that testosterone improved AMS scores in a group with initial testosterone ≤8.0 nM but not in a group with initial testosterone 8.1–12 nM.

Ten of 13 of the studies on libido or desire with a Jadad score of 4 or 5 found a benefit. Seven of 12 studies on erectile dysfunction with a Jadad score of 4 or 5 found a benefit.

### 3.3 Muscle Weakness/Wasting

Table 3 summarizes 39 studies that evaluated the effect of testosterone on physical function, muscle strength, or HIV-associated muscle wasting, including 19 in men assessed as having low serum testosterone, 9 on HIV-negative men with normal serum testosterone, 1 on healthy men with normal serum testosterone, and 10 on HIV-positive men. Studies that measured testosterone effects only on body composition (other than in HIV-associated wasting) without measures of physical function or muscle strength were excluded. Subjects included both those defined by the authors as hypogonadal and those considered to have normal testosterone concentrations. Common measures of muscle strength included grip strength dynamometry and the 1-repetition maximum for exercises including the bench press and leg press. Physical function was often measured by the 6-minute walk test, the time and number of steps required to walk 25 feet, and the get-up-and-go test, which evaluates the ability to rise from a chair, walk a short distance, and return to sitting.

**Table 3:** Effects of Testosterone on Muscle Weakness/Wasting.

Men Assessed as Having Low Serum Testosterone				
**Brill et al, 2002**[92]	10 men 60–78 years old with morning serum T 200–450 ng/dL (6.9–15.6 nM), serum prolactin below 25 μg/L, LH and FSH below 20 IU/L, IGF-I below 200 μg/L.	T patch 5 mg/day or placebo x 1 month with each subject serving as his own control; subjects also received growth hormone with or without T (not discussed here); randomization method not given.	No change in eccentric/concentric knee extension/flexion strength, hamstring flexibility, or eyes-closed non-dominant-leg balance test. ↑Fat-free mass by 1 of 2 measurement techniques. No change in % body fat or BMI.	3
**Casaburi et al, 2004**[84]	53 men 55–80 years old with COPD (FEV_1_ ≤60% of predicted, FEV_1_: VC ratio ≤60%) and serum T ≤400 ng/dL (13.9 nM); 6 dropouts were excluded from analysis.	T enanthate 100 mg (n = 23) or placebo (n = 24) IM weekly with or without resistance training x10 weeks; randomization method not given.	T + no training group: Compared to placebo + training: ↑trunk lean mass, ↓% fat. Compared to placebo groups: ↑arm and total lean mass; ↓leg and total fat. Compared to placebo + no training: ↑leg lean mass and leg press strength; ↓leg press fatigue and peak work rate. No change: arm and trunk fat, maximal inspiratory pressure, peak O_2_ uptake, lactic acidosis threshold, constant work rate duration.	5
			T + strength training group: Compared to placebo groups: ↑arm, trunk, leg, and total lean mass; ↓leg, total, and % fat.	
			Compared to non-training groups: ↑leg press strength and peak oxygen uptake; ↓leg press fatigue. Compared to T + no training: ↑peak work rate. Compared to placebo + no training: ↑lactic acidosis threshold. No change: arm and trunk fat, maximum inspiratory pressure, constant work rate duration.	
**Clague et al, 1999**[165]	14 men mean age 68.1 with total T <400 ng/dL (13.9 nM).	T enanthate 200 mg (n = 7) or placebo (n = 7) IM every 2 weeks x 12 weeks, with muscle testing every 4 weeks; randomization method not given.	No change in handgrip, knee extensor, and knee flexor strength, leg extensor power, step height.	4
**Emmelot-Vonk et al, 2008**[91]	237 men 60–80 years old with total T <395 ng/dL (13.7 nM); 30 dropouts were excluded from analysis.	T undecanoate 80 mg (n = 120) or placebo (n = 117) capsules twice daily x 6 months; a randomization list without stratification using blocks of 6 was computer-generated using the ADLS.	↓Total body fat mass and body fat percentage; ↑total body lean mass. No change in grip strength, leg extensor strength, timed get up and go test.; No change in BMI, intra-abdominal fat mass.	5
**Ferrando et al, 2001**[93]	12 healthy men, mean age 67–68 with serum total T ≤480 ng/dL (16.7 nM).	T enanthate (n = 7) or placebo (n = 5) IM (dosage adjusted to maintain nadir serum T concentrations 17–28 nM) weekly x 1 month, then every 2 weeks x 6 months; randomization method not given.	No change at 1 month in strength. At 6 months: ↑strength for bicep curl, tricep extension, leg extension. No change in leg-curl strength and knee extension endurance in the dominant leg. ↑Total lean body mass, leg lean mass, leg muscle volume; ↓fasting protein breakdown % body fat. No change in arm lean mass	0
**Hildreth et al, 2013**[94]	167 men over 60 years old (mean age 66) with total T 200–350 ng/dL (6.9–12.1 nM); 24 dropouts were excluded from analysis.	T gel 1% 2.5, 5.0 g (n = 111), or placebo (n = 56) daily x 12 months, with or without progressive resistance exercise training; randomization was performed using permuted block randomization with random block sizes.	T treated groups analyzed in aggregate. In subjects with resistance training, no change in physical functional performance tests: upper body strength and flexibility, lower body strength, balance, and endurance, stair climb speed and 6-min walk, strength (bench press, incline press, overhead pull-down, seated row, average upper body, grip strength, knee extension, knee flexion, seated leg press, average lower body), power (leg extensor). ↓Fat mass; ↑overall and arm fat free mass. No change in weight, BMI, trunk fat mass, appendicular and leg fat-free mass, waist and hip circumferenceIn subjects without PRT: ↑Lower body strength, strength for bench and incline press, average upper body and grip strength. No change in physical functional performance test total score, upper body strength and flexibility, lower body balance and endurance, stair climb speed and 6-min walk, strength for overhead pull-down, seated row, knee extension/flexion, seated leg press, and average lower body, power (leg extensor). ↑Appendicular and arm fat free mass. ↓Overall and trunk fat mass, waist circumference. No change in weight, BMI, overall and leg fat-free mass, hip circumference	5
**Kenny et al, 2001**[166]	67 men >65 years old (mean age 76) with bioavailable T <128 ng/dL (4.44 nM); 23 dropouts were excluded from analysis.	T patch 5 mg (n = 34) or placebo (n = 33) daily x 1 year; all received 500 mg calcium and 400 IU vitamin D supplements; randomization method not given.	No change in BMI, % body fat or lean mass. No change in PASE (Physical Activity Scale for the Elderly) score. PASE includes 8-ft walk speed, chair rise, single-leg stance, supine-to-stand, and get up and go time.	4
**Kenny et al, 2010**[80]	131 men ≥60 years old (mean age 77.1) with T <350 ng/dL (12.1 nM), physical frailty, and BMD T-score at the hip ≤ —2.0 or a nontraumatic fracture within last 5 years; delays in recruitment and lack of funds resulted in ≥16 months of follow-up with analysis performed at 12 months; 69 dropouts excluded from analysis.	T gel 5 mg (n = 69) or placebo (n = 62) daily x 16 months; all subjects maintained calcium intake of 1500 mg/day and were given 1000 IU cholecalciferol/day; randomization with block sizes (2 or 4) stratified by frailty status	↑Total body lean mass, appendicular skeletal muscle mass; No change in total body fat %. No change in hand grip/ leg press strength or PASE	5
**Ly et al, 2001**[11]	37 healthy men >60 years old (mean age 68.2) with plasma T ≤429 ng/dL (14.9 nM); 4 dropouts were excluded from analysis or for some measures, missing data imputed using last observation carried forward	0.7% DHT gel 70 mg (n = 17) or placebo (n = 16) daily x 3 months, followed by 1 month of observation; method of randomization inadequately explained	↑Dominant knee flexion peak torque from 2–4 months. No change in waist:hip ratio, lean mass, peak torque for knee extension, nondominant knee flexion, shoulder flexion/extension, maximal horizontal forward reach of the outstretched right arm without losing balance; standing balance time with feet tandem, eyes closed, 18 meter fast walk, time to stand up and sit 5x. ↓Body weight (month 4), fat mass (months 2–4), skinfold thickness (months 2–4).	5
**Nair et al, 2006**[95]	62 men >60 years old with bioavailable T <3.6 nM (104 ng/dL); incomplete follow-up for some subjects, 4 subjects did not complete study.	Transdermal T patch 5 mg (n = 30) or placebo (n = 32) x 24 months; randomization method not given. Additional arms included women and included other tx (not discussed here)	No change in peak VO_2_, peak torque of isometric dominant knee extension, strength for seated chest press and double leg press. ↑Fat-free mass. No change in body weight, BMI, body fat %, visceral:total fat ratio, visceral fat, thigh-muscle area	4
**Okun et al, 2006**[85]	30 men mean age 68 with Parkinson disease and free T <100 pg/mL (347 pM).	200 mg T enanthate (n = 15) or placebo (n = 15) IM every 2 weeks x 8 weeks; dose could be adjusted upwards based on free T measured every 2 weeks; randomization method not given.	No change in motor function as measured by a videotaped Unified Parkinson’s Disease Rating Scale (UPDRS) evaluation.	4
**Page et al, 2005**[81]	48 men age 65–83 (mean 71) with morning serum total T <350 ng/dL (12.1 nM), classified as sedentary (≤60 min/week of moderate-intensity recreational physical activity); 13 dropouts excluded from analysis	T enanthate 200 mg (n = 24) or placebo (n = 24) IM every 2 weeks x 36 months; subjects also received T with finasteride (not discussed here); randomization method not given	↑Improvement in timed physical performance, right handgrip strength. No change in body weight, left handgrip strength, peak torques of knee/ankle flexion/extension. ↓Total fat mass, % body fat, right leg fat, trunk fat. ↑Lean body mass, waist:hip circumference ratio	4
**Sih et al, 1997**[104]	32 men (mean age 68 for placebo and 65 for T) with bioavailable blood T ≤60 ng/dL (2.1 nM); 3 T subjects dropped due to an abnormal increase in hematocrit of ≥52%	T cypionate 200 mg (n = 17) or placebo (n = 15) IM every 14–17 days x 12 months; randomization by random number	↑Grip strength. No change in body weight, BMI	4
**Srinivas-Shankar et al, 2010**[53]	297 men ≥65 years old (mean age 73) with physical frailty and total T ≤345 ng/dL (12 nM); 31 dropouts excluded from analysis	T 50 mg (n = 136) or placebo (n = 138) gel daily x 6 months; dosage adjusted if T concentrations remained outside the target range (18–30 nM); subjects randomized in blocks of 10 by computer-generated sequence	↑Isometric knee extension peak torque↑LBM, ↓fat mass. No change in isokinetic knee extension peak torque, isometric/ isokinetic knee flexion peak torque, grip strength, aggregate locomotor function (ALF) test, physical performance test (PPT), 6 minute walk, PASE, Tinetti gait/balance test.	5
**Snyder et al, 1999**[96]	108 men >65 years old with serum T <495 ng/dL (17.2 nM); 12 dropouts were excluded from analysis.	T patch 6 mg (n = 54) or placebo (n = 54) daily x 36 months; every 3 months T concentration was checked and dosage was decreased to 4 mg/day if serum T was >1000 ng/dL; randomization method not given.	No change in weight, BMI, tissue mass, trunk fat mass, arm/leg lean mass, strength for knee extension/ flexion and handgrip, time to walk and number of steps in 25 feet, time to walk 12 stairs. ↓Fat mass, arm/leg fat mass. ↑Lean mass, trunk lean mass	4
**Sullivan et al, 2005**[82]	71 men ≥65 years old (mean age 78.2) with serum total T <480 ng/dL (16.7 nM) and a recent functional decline; 10 dropouts were excluded from analysis.	T enanthate 100 mg (n = 37) or placebo (n = 34) IM weekly with low- or high-intensity resistance strength training x 12 weeks; randomization stratified by arbitrary score of physical ability; within each stratum, subjects randomized with blocks sizes in multiples of 4 and randomly varied	No change in strength for chest and leg press, performance testing score (sit-to-stand maneuver, habitual and maximal safe gait speed tests, stair climb). ↑Mid-thigh cross-sectional muscle area	5
**Travison et al, 2011**[83]	209 men ≥65 years old with total serum T 100–350 ng/dL (3.5–12.1 nM) or free serum T <50 pg/mL (174 pM) with mobility limitations; analysis restricted to 165 men with a baseline assessment and at least one outcome assessment.	T 100 mg (n = 106) or placebo (n = 103) gel daily x 6 months; after 2 weeks, dose level was increased or decreased by 50% based on serum T; randomization by computer-generated table in blocks of 6 stratified by age	↑Strength for leg and chest press, loaded stair-climbing power, loaded walk speed. No change in grip strength, unloaded walking speed, unloaded stair-climbing power. ↑Total lean mass, appendicular skeletal muscle mass. ↓Total fat mass, appendicular fat mass. ↑Cardiovascular-related AEs with T (23 vs 5)	5
**Wittert et al, 2003**[97]	76 healthy men age ≥60 (mean 68.5) with ≥2 symptoms on the ADAM questionnaire, a ratio of T/sex-hormone binding globulin of 0.3–0.5, serum total T >230 ng/dL (8 nM); 18 dropouts were excluded from analysis.	T undecanoate 80 mg (n = 39) or placebo (n = 37) twice daily x 12 months; randomization with blocks of 4 as part of a proprietary system	No change in grip, quadriceps, and calf strength;No change in body weight. ↑Lean body mass; ↓% body fat	5
**Borst et al, 2014**[167]	60 men ≥60 years old with serum testosterone concentration ≤300 ng/dL (10.4 nM) or bioavailable testosterone ≤70 ng/dl (2.4 nM). 40 completed. Randomized by computer program	4 groups: T enanthate 125 mg/ week IM X 52 weeks and finasteride 5 mg/day; T enanthate and oral placebo; vehicle i.m and finasteride, or vehicle IM and placebo.	Testosterone, compared to finasteride or placebo, increased strength (leg press, knee flexion and extension, chest press, triceps extension, and grip strength), body fat-free mass. Total fat mass reduced 3.87 kg. Lumbar spine and hip BMD increased	5
**HIV-Negative Menwith Normal Serum Testosterone**				
**Amory et al, 2002**[86]	25 men 58–86 years old undergoing knee replacement surgery; 3 dropouts were excluded from analysis.	T enanthate 600 mg (n = 10) or placebo (n = 12) IM 21, 14, 7, and 1 day before surgery; randomized using a random number sequence.	↑Standing function at post-operative day 3. No change in standing function at post-operative day 35, walking and stair climbing tests, length of hospital stay	5
**Bakhshi et al, 2000**[90]	15 men ≥65 years old who had been admitted to the Geriatric Evaluation and Management unit for rehabilitation; 1 subject in the T group died unexpectedly 2 weeks after admission on the day of his planned discharge probably as the result of a cardiovascular event.	T enanthate 100 mg (n = 9) or placebo (n = 6) IM weekly until discharge or for a maximum of 8 weeks; subjects received rehabilitation therapy as appropriate to their needs; randomization method not given.	↑Functional Independence Measure and grip strength compared to baseline; difference compared to placebo not reported, but no difference in either endpoint compared to placebo by *t*-test performed by us.	4
**Caminiti et al, 2009**[18]; **Schwartz et al, 2011**[87]	70 men 66–76 years old (mean age 70) with stable CHF (NYHA II or III) and LV EF <40%; 6 dropouts (4 on T and 2 on placebo) were excluded from analysis.	T undecanoate 1000 mg (n = 35) or saline (n = 35) IM at 6 and 12 weeks. Subjects said to be randomized, randomization method not given	↑Peak VO_2_, peak workload, distance walked in 6 minutes, leg muscle strength, knee extension/flexion peak torque. ↓Ventilation/CO_2_ output. ↑Body weight, BMI	4, 5
**Dohn et al, 1968**[13]	44 men with leg claudication or ulcers attributed to arteriosclerosis in 43 men and to Buerger’s disease in 1 man; age not given. Two men did not complete study. Numbers in tables add to 86 subjects. Not possible to tell for sure how many men were analyzed.	Aqueous T isobutyrate 300 mg or meprobamate as placebo every 14 days for 3 months; route of administration not given; double-blinded, randomization not discussed	No effect on subject improvement, walking test	3
**Crawford et al, 2003**[88]	43 men >20 years old (mean age 60.3) on long-term glucocorticoid therapy; 14 dropouts were excluded from analysis.	T mixed esters 200 mg (n = 18) or placebo (n = 16) IM every 2 weeks x 12 months; all received calcium carbonate 600 mg daily; randomization method not given. Nandrolone not discussed.	↑Peak knee extension/flexion torque. ↑Lean mass. ↓Fat mass. No change in body weight, BMI, waist circumference, waist:hip ratio, skinfold thickness.	3
**Griggs et al, 1989**[89]	40 men 18–65 years old (mean age 33.3 years for T and 41.5 years for placebo) with myotonic dystrophy and a typical distribution of weakness (but ambulatory); 3 dropouts were excluded from analysis.	T enanthate or placebo 3 mg/kg IM weekly x 12 months; number of subjects/group not given; evaluations every 3 months; randomization performed using computer-generated random numbers, stratified by grip strength and previous T tx	↑Time to climb stairs. No change in arm volume, weight, manual muscle testing, myometry, grip dynamometry, forced vital capacity (VC), maximum voluntary ventilation, maximum expiratory pressure, time to walk 30 feet, time to cut standard square. ↑Creatinine excretion (↑muscle mass)	4
**Hentzer & Madsen, 1967**[40]	39 males with arterial insufficiency; 3 drop outs.	200 mg T (n = 19) or placebo (n = 17) IM weekly for 3 weeks, then once every second week for 6 months; consecutive patients assigned to tx or placebo by record numbers.	No change in grip strength, walking distance.	3
**Svartberg et al, 2004**[43]	29 men 54–75 years old (mean age 66 years) with moderate to severe COPD; 2 dropouts were excluded from analysis.	T enanthate 250 mg (n = 15) or placebo (n = 14) IM every 4 weeks x 26 weeks; method of randomization not discussed	No change in BMI, forced VC, forced expiratory volume, 6-minute walk. ↑Fat-free mass. ↓Total body fat mass (at 12 weeks).	4
**Mirdamadi et al, 2014**[22]	50 males age 50–70 with CHF	T enanthate 250 mg IM or saline placebo IM every 4 weeks for 12 weeks	No difference between groups in muscle strength or body weight. Both groups improved in 6-minute walking distance; no post-intervention effect was seen.	3
**Giannoulis et al, 2006**[168]	Healthy men 65–80 years old with circulating IGF-I <50^th^ percentile for the local age-specific range; of those meeting criteria, the 80 subjects with the lowest T concentration but without T deficiency, mean 397.6 ng/dL (13.8 nM); 5 subjects were excluded because PSA concentrations met revised exclusion criteria and 6 dropouts were excluded from analysis.	T 5 mg or placebo transdermal patch daily x 6 months; randomization was performed using computer-generated pre-allocated study numbers; growth hormone arm not discussed here	No change in knee flexion/extension peak torque/force, hand-grip peak force, VO_2_ max. No change in BMI, hip/waist ratio, lean body mass, subcutaneous/cross-sectional abdominal visceral/total body fat, mid-thigh muscle cross sectional area.	5
**HIV-Positive Men**				
**Bhasin et al, 2007**[98]	88 HIV-positive men 18–70 years old with abdominal obesity and serum total T 125–400 ng/dL (4.3–13.9 nM); 8 dropouts were excluded from analysis.	Transdermal T gel 100 mg (n = 44) or placebo (n = 44) daily x 24 weeks; randomization stratified by RNA copy number with approximate balance within each site	↓Total abdominal, subcutaneous abdominal, trunk, extremity, and whole body fat mass; waist circumference; waist:hip ratio. ↑Trunk, extremity, and total lean mass. No change in visceral fat area, body weight, BMI	5
**Bhasin et al, 1998**[103]	41 HIV-positive men 18–60 years old with serum T <400 ng/dL (13.9 nM); 9 dropouts were excluded from analysis.	T patch 5 mg (n = 20) or placebo (n = 21) daily x 12 weeks; randomization method not given	No change in strength (squat and bench press); ↓Fat mass. No change in lean body mass (LBM), fat-free mass, total body weight	3
**Bhasin et al, 2000**[99]	61 HIV-positive men 18–50 years old with involuntary weight loss, serum total T <349 ng/dL (12.1 nM); 12 dropouts were excluded from analysis.	T enanthate 100 mg (n = 32) or placebo (n = 29) IV x 16 weeks with or without resistance exercise; randomization by random number table stratified by age in blocks of 16.	T with no-exercise group: no change in strength for leg press, leg curls, bench press, latissimus pulls, overhead press; ↑Body weight, muscle volume; no change in fat-free and fat mass, total body water (TBW), TBW:fat-free mass ratio, LBM of arms/legs/trunk. T with exercise group: no changes in any measure	4
**Coodley et al, 1997**[105]	39 HIV-positive men 18–60 years old with AIDS and weight loss; 4 dropouts were excluded from analysis.	T cypionate 200 mg (n = 17) or placebo (n = 18) IM every 2 weeks x 3 months followed by tx switch x 3 months; randomization method not given	No change in weight, triceps and scapula skinfold	3
**Dobs et al, 1999**[113]	133 men with AIDS age 18–65+ who had lost 5–20% of baseline weight with a morning serum total T concentration ≤400 ng/dL (13.9 nM) or free T concentration ≤16 pg/mL (56 pM); 15 T and 20 placebo subjects did not complete.	T 15-mg (n = 67) or placebo (n = 66) patch applied 20–24 hours/day to shaved scrotal skin for 12 weeks; assessments at weeks 4, 8, and 12; randomization method not given	No effect on body cell mass or weight.	3
**Fairfield et al, 2001**[107]	54 HIV-positive men (mean age 38 years) with normal serum free T >12 pg/dL (0.4 pM) and wasting; 11 dropouts were excluded from analysis.	T 200 mg (n = 24) or placebo (n = 26) IM weekly with or without progressive resistance training x 12 weeks; patients were stratified by body weight, but randomization method not given	↑Thigh muscle attenuation on computerized tomography (CT) only in subjects who received training. Strength testing was evaluated only with respect to attenuation by CT, not tx group.	4
**Grinspoon et al, 1998**[100]	51 HIV-positive men mean age 42 with free T <11.9 pg/dL (0.4 pM) and wasting; 11 dropouts were excluded from analysis.	T enanthate 300 mg (n = 21) or placebo (n = 19) IM every 3 weeks x 6 months; subjects stratified by body weight and megestrol acetate use; randomization using a permuted block algorithm	No change in fat mass, TBW, exercise performance (6-minute walk test, timed sit-to-stand test, timed get-up-and-go test). ↑Fat-free mass, LBM, muscle mass, total body potassium content	5
**Grinspoon et al, 2000**[101]	54 HIV-positive men with wasting and serum free T >42 pM (1.2 ng/dL); 11 dropouts were excluded from analysis.	T enanthate 200 mg (n = 24) or placebo (n = 26) IM weekly x 12 weeks with progressive strength training + aerobic conditioning or no training; randomization with permuted-block algorithm (blocks of 8).	↑Peak isometric force for shoulder extension and elbow flexion. No change in peak isometric force for knee flexion/extension and dorsiflexion. Grip strength results were not reported. ↑Weight, LBM, arm muscle area, leg muscle area	5
**Knapp et al, 2008**[41]	61 HIV-positive men 18–60 years old (mean age 43) with unintentional weight loss or BMI <20 and serum T <400 ng/dL (13.9 nM); 4 placebo-assigned and 9 T-assigned subjects dropped out and were analyzed using the last observation carried forward.	T enanthate 300 mg (n = 30) or placebo (n = 31) IM weekly x 16 weeks; computer-generated randomization list with a block size of 6	No change in physical function measures (stair-climbing power, walking speed, load-carrying ability), muscle performance (leg press strength, leg press power, leg press fatigability). ↑Fat-free mass. No change in fat mass, total weight, % body fat.	5
**Sardar et al, 2010**[106]	104 HIV-positive men >18 years old with involuntary weight loss or BMI <20; randomized subjects evaluated using ITT analysis.	Sustanon 250 = T propionate 30 mg, phenylproprionate 60 mg, isocaproate 60 mg, and decanoate 100 mg (n = 42) or placebo (n = 20) IM every 2 weeks x 12 weeks; computer-generated randomization tables in a 2:1 ratio	↑Weight. No change in BMI, waist and hip circumference, waist:hip ratio, triceps and scapula skinfold thickness, mid-arm circumference, % body fat	5

Twenty studies evaluated subjects described as hypogonadal, with 11 of those evaluating healthy subjects. Five studies examined the effects of testosterone supplementation on physical frailty, functional limitations, or a categorization as “sedentary,”[53, 80–83] and single studies evaluated subjects with COPD,[84] advanced cancer,[75] and Parkinson’s disease.[85] Ten studies evaluated subjects considered to have normal testosterone concentrations; 1 study included healthy, elderly men, and the remainder included subjects with planned knee replacement surgery,[86] stable CHF,[18, 22, 87] leg claudication or ulcers,[13] long-term glucocorticoid therapy,[88] myotonic dystrophy,[89] arterial insufficiency,[40] COPD,[43] or who were planning or undergoing physical rehabilitation.[90]

Ten studies evaluated subjects with HIV; 8 of those studies included subjects with HIV-wasting, 1 included subjects with abdominal obesity, and 1 did not use weight criteria. Most of these papers studied older men. Few studies investigated the use of testosterone supplementation in men younger than 60 years.

Twenty-seven studies measured the effects of testosterone treatment on muscle mass, with 22 (81%) of these studies showing a significant increase in muscle mass associated with treatment.[41, 43, 53, 80–84, 88, 89, 91–102] Nineteen of 22 (86.3%) of these studies had a Jadad score of 4 or 5. Twenty-five studies assessed the effects of testosterone treatment on fat mass, with 15 (60%) of these studies showing a decrease in fat mass associated with treatment.[11, 43, 53, 81, 83, 84, 88, 91, 93, 94, 96, 97, 99, 102, 103] Twelve of these studies had a Jadad score of 4 or 5.

Some studies did not measure muscle and fat mass specifically but used other body composition endpoints. Two studies showed no changes in body weight or BMI,[22, 104] but another showed an increase in body weight and BMI.[18] One study, with a Jadad score of 3, showed no change in weight or estimates of body fat (triceps and scapula skinfold thickness).[105] In studies of HIV-positive men with weight loss, 3 of 6 studies (all of which had Jadad scores of 4 or 5) showed an increase in weight with testosterone treatment,[99, 101, 106] and all 4 studies that measured muscle mass showed an increase.[41, 99–101]

Of the 30 studies that assessed muscle strength as a primary or secondary endpoint, 13 studies (43%) reported an improvement in at least 1 measure of muscle strength.[11, 18, 53, 81, 83, 84, 88, 93, 94, 101, 102, 104, 107] Eleven of 13 of these studies had a Jadad score of 4 or 5. Three of these 12 studies (all with Jadad scores of 4 or 5) reported improvements in fewer than 25% of the measurements.[11, 53, 81] In studies of men without HIV, 11 of 24 studies (45.8%) reported an improvement in at least 1 measure of muscle strength. In studies of men with HIV, 2[101, 107] of 5 studies reported an improvement in at least 1 measure of muscle strength; 3 showed no effect.[41, 99, 103]

Twenty-four studies evaluated the effects of testosterone treatment on physical function endpoints and, of these, 5 found an improvement in at least 1 measure of function.[18, 22, 81, 83, 86] Neither of the 2 studies of HIV patients measuring physical function showed an improvement in function.[41, 100] Six of these studies had a Jadad score of 4 or 5.[18, 41, 81, 83, 86, 100]

In summary, the majority of studies show increased muscle mass but no effect of testosterone on muscle strength or function.

### 3.4 Mood and Behavior

Forty-five studies evaluating the effect of testosterone on mood and behavior are summarized in Table 4. Twenty-nine of these studies focused on men without psychiatric disorders, and 16 on men with psychiatric disorders.

**Table 4:** Effects of Testosterone on Mood and Behavior.

**Menwithout Psychiatric Disorders**				
**Skakkebaek et al, 1981**[45]	12 men 22–48 years old diagnosed as androgen deficient; one man subsequently found to be normal and excluded.	T undecanoate 160 mg or placebo daily for 2 months followed by opposite tx; randomization of tx order not described but said to be balanced.	Improved self-rated anxiety and tension; no statistically significant effect on depression, anger, vigor, or fatigue (authors concluded otherwise based on *P* = .10).	3
**Anderson et al, 1992**[63]	31 healthy men 21–41 years old.	T enanthate 200 mg IM weekly for 8 (n = 16) or 4 weeks with placebo IM weekly for the remaining 4 weeks (n = 15); randomization not discussed. Described as single-blinded.	No effect on self-assessment of mood (cheerful, lethargic, relaxed, tense, energetic, unhappy, irritable, ready to fight, easily angered) using an unvalidated ranking scale.	2
**Janowsky et al, 1994**[114]	56 healthy men 60–75 years old.	T 15-mg scrotal (n = 27) or placebo patch (n = 29) 16 hours daily for 3 months; method of randomization not discussed.	No effect on self-rated or spouse-rated Profile of Mood States (POMS).	3
**Kouri et al, 1995**[108]	8 healthy men 20–39 years old; 2 were subsequently excluded from analysis because they did not accept the terms of the test situation.	Crossover design: 1) T cypionate IM 150 mg/week for 2 weeks, then 300 mg/week for 2 weeks, then 600 mg/week for 2 weeks then no tx for 6 weeks, then placebo IM for 6 weeks, then no tx for 6 weeks or 2) Same design with placebo tx prior to T.	↑Aggressive response to a button-pushing game involving retaliation against a fictitious opponent. ↑Young Mania Rating Scale score (more aggressive).	4
**Bhasin et al, 1996**[115]; **Tricker et al, 1996**[116]	50 healthy men 19–40 years old who had earlier experience with weight-lifting; 7 dropped out prior to randomization, 3 dropped out during tx and were excluded.	T enanthate 600 mg or placebo IM weekly followed by the opposite tx, tx for 10 weeks followed by washout. Further randomization within tx to exercise or non-exercise. Randomization method not given.	No effect on Multidimensional Anger Inventory, Mood Inventory, or Observer Mood Inventory administered prior to tx and during weeks 6 and 10, 7 days after previous injection.	2, 4
**Schiavi et al, 1997**[59]	18 men 46–67 years old (median age 60 years) with ED with or without hypoactive sexual desire; 12 men completed the study, dropouts were excluded from analysis.	T enanthate 200 mg or placebo IM every 2 weeks for 6 weeks followed by 4-week washout followed by the opposite tx; order was randomized by unstated method; 7 subjects received placebo first, 5 subjects received T first.	No effect on POMS or psychological symptom profile.	3
**Sih et al, 1997**[104]	32 men 51–79 years old with free blood T concentration <60 ng/dL (2.1 nM); unclear how dropouts after randomization were handled.	T cypionate 200 mg (n = 10) or placebo (n = 12) every 14–17 days for 12 months; tx allocation by random number (not otherwise characterized)	No effect on Yesavage Geriatric Depression Scale (GDS)	4
**Dobs et al, 1999**[113]	133 men with AIDS age 18–65+ who had lost 5–20% of baseline weight with a morning serum total T concentration ≤400 ng/dL (13.9 nM) or free T concentration ≤16 pg/mL (56 pM); 15 T and 20 placebo did not complete the study.	T 15-mg (n = 67) or placebo (n = 66) patch applied 20–24 hours/day to shaved scrotal skin for 12 weeks; assessments at weeks 4, 8, and 12; randomization method not given.	No effect on health distress or mood assessed by Rand HIV-Medical Outcomes Study-short form or EUROQoL Feeling Thermometer	3
**Pope et al, 2000**[169]	53 presumed healthy men aged 20–50 years; an additional 13 men were recruited but not randomized or were randomized and not evaluable.	T or placebo IM every 2 weeks. T dose was 150 mg/week for 2 weeks, 300 mg/week for 2 weeks, and 600 mg/week for 2 weeks. After a 6-week washout period, opposite tx given. Method of randomization not given	↑Young Mania Rating Scale (↑manic).↑Point Subtraction Aggression Paradigm (↑aggressive). ↑Verbal hostility subscale score on Aggression Questionnaire of Buss and Perry. ↑Phobic anxiety on Symptom Checklist 90-R. No effect on Hamilton Depression Rating Scale (HAM-D)	4
**Daly et al, 2001**[109]	20 healthy men aged 18–42 years.	PO placebo daily for 3 days, then PO methyltestosterone 40 mg/day for 3 days, then PO methyltestosterone 240 mg/day for 3 days, then PO placebo for 3 days inpatient. Schedule fixed but unknown to subjects and raters (not clear who the raters were inasmuch as subjects self-rated).	High-dose methyltestosterone associated with visual analogue scale (VAS) self-rating of ↑Cognitive ability, ↑Distractibility, ↑Energy, ↑Sexual arousal, ↑Aggression, ↑Irritability.	2
**O’Connor et al, 2002**[117]	30 healthy eugonadal men aged 23–40 years; 8 hypogonadal men included but given only active tx and so not summarized here. One subject dropped out; it is not clear if he was retained in the analysis.	Testosterone enanthate 200 mg IM or placebo weekly for 8 weeks (n = 15/group). Method of randomization not given	The authors reported a lack of effect of testosterone on mood and aggression in eugonadal men, but the analyses shown in the paper were chiefly between hypogonadal men and eugonadal men.	2
**Dabbs et al, 2002**[110]	16 healthy men and 17 women, mean age 20.2 years (women are not further discussed); 5 subjects (sex not indicated) failed screening; 15 men were scored.	Micronized T 40 mg or placebo gel applied to the skin daily for 5 days	No difference in personality scores on Gough and Heilbrun Adjective Check List; some differences identified by study authors on *post-hoc* analysis. ↑Hostility on evaluation by 2 undergraduate judges of a free-text paragraph written by each subject on his mood at the end of tx	3
**Kunelius et al, 2002**[118]	120 men, age 50–70 years, with “andropause” symptoms, serum T <15 nM (432 ng/dL). Six subjects dropped out; it is not clear whether they were included in the analysis.	“Randomized” by sealed envelope to 2.5% DHT gel (125 mg/day DHT) or placebo (60 subjects per group) for 30 days after which dose was adjusted on serum DHT concentration to 187.5 or 250 mg/day DHT. Placebo dose was randomly adjusted. Tx duration was 6 months.	No effect on well-being or mood as assessed by questionnaire.	5
**O’Connor et al, 2004**[67]	28 healthy eugonadal men 22–44 years old; 4 subjects withdrew on tx and were excluded.	T undecanoate 1000 mg or placebo IM at the beginning of an 8-week tx phase followed by 8-week washout followed by the opposite tx. Self-assessment each week and psychometric assessments during week 4 of each tx phase	↑Anger-hostility (during first 2 weeks after injection) and ↓fatigue-inertia scores on POMS. No effect on Aggression Questionnaire, Partner Aggression Questionnaire, or Aggression Provocation Questionnaire, Buss Durkee Hostility Inventory (irritability subscale), Rathus Assertiveness Schedule, or State Self-Esteem Scale	3
**Howell et al, 2001**[24]	35 men mean age 40.9 with some degree of testicular dysfunction after cytotoxic cancer therapy; blood LH ≥8 mIU/L and T <20 nM (576 ng/dL); 2 dropouts were excluded from analysis.	T 2.5 or 5.0 mg patch (n = 16) or placebo patch (n = 19) daily for 12 months; randomization method not described, study described as single-blinded	No effect on Hospital Anxiety and Depression Scale (HADS).	3
**Pugh et al, 2004**[20]	20 men 44–81 years old with impaired LV EF (mean 35%)	T 100 mg or placebo IM every 2 weeks for 12 weeks. Subjects said to be randomized, but randomization method and the number of subjects per group were not described.	BDI not affected; improvement identified by authors was not statistically significant.	3
**Haren et al, 2005**[119]	76 men 60–86 years old, mean age 68.5 years, with at least 2 symptoms on ADAM and total T >8 nM (231 ng/dL); 6 dropouts analyzed by ITT	T undecanoate 160 mg/day (n = 39) or placebo (n = 37) by mouth for 12 months; dose halved if hematocrit increased above 50%; randomization in blocks of 4 using ADLS	More GDS improvement in placebo vs T group	5
**Malkin et al, 2006**[23]	76 men with CHF, mean age 64 years; 34 dropouts were retained for analysis using ITT.	T 5 mg (n = 37) or placebo (n = 39) patch daily for 12 months; randomization was stratified by ischemic vs non-ischemic heart failure. Method of randomization not given	No effect on BDI	3
**Brockenbrough et al, 2006**[38]	40 hemodialysis patients with serum T concentration <400 ng/dL (13.9 nM); 22 subjects completed the study. Analyzed by ITT	Topical T 100 mg (n = 19) or placebo gel (n = 21) daily for 6 months; block randomized in groups of 4	Not possible to evaluate; Likert scale for mood combined with scales for sexual desire and reported as a composite score not affected by tx	5
**Okun et al, 2006**[85]	Men with Parkinson disease and free T concentration <100 pg/mL (347 pM), mean age 68 years	T enanthate 200 mg (n = 15) or placebo (n = 15) IM every 2 weeks for 8 weeks; dose could be adjusted upwards based on free T concentration measured every 2 weeks; method of randomization not given	No effect on GDS, State-Trait Anxiety Inventory (STAI), or Visual Analog Mood Scale (VAMS)	4
**Knapp et al, 2008**[41]	61 HIV-positive men, mean age 43 years, with involuntary weight loss and/or BMI <20 mg/m^2^; 4 placebo-assigned and 9 T-assigned subjects dropped out and were analyzed using the last observation carried forward.	T enanthate 300 mg (n = 31) or placebo (n = 30) weekly for 16 weeks; randomized by computer-generated list in blocks of 6	↑Mental health subscale of QoL instrument. ↓Stress, anxiety, and depression subscale scores	5
**Maki et al, 2007**[120]	15 men age 66–86 years, total blood T >240 ng/dL (8.3 nM)	T enanthate 200 mg (n = 9) or placebo (n = 6) IM every other week for 90 days followed by 90-day washout followed by the opposite tx; tx order determined by coin toss	No tx effect on Positive and Negative Affectivity Schedule	5
**Vaughan et al, 2007**[112]	69 men 65–83 years (mean 70.8 years) with 2 morning serum T concentrations <12.1 nM (350 ng/dL); 23 dropouts were excluded from analysis.	T enanthate 200 mg IM every 2 weeks + placebo pill (n = 23), T enanthate 200 mg IM every 2 weeks + finasteride 5 mg/day (n = 22), or placebo injection and placebo pill (n = 23), tx for 36 months; block-randomization by computer; possible reduction in T dose if hematocrit >52%; cognitive tests at baseline, 4, and 36 months	↓Scores in Speilberger Test of Anxiety. No effect on BDI	5
**Zak et al, 2009**[122]	25 male students, mean 20.8 years old.	T 1% gel 10 g or placebo given prior to playing game; each man served as his own control. Randomization of tx order not discussed	↑Selfishness in game designed to test selfishness	4
**Giltay et al, 2010**[75]	184 men age 35–69 (mean 52.1) with T concentration <12.0 nM (346 ng/dL) or calculated free T concentration <225 pM (6.5 ng/dL) who had metabolic syndrome; 14 dropouts (8 T, 6 placebo) analyzed with last observation carried forward	T undecanoate 1000 mg (n = 113) or placebo (n = 71) IM given at week 0, 6, 18, and 24; evaluations at week 30. Randomization method not given except to say that T was over-assigned in a 7:3 ratio	↑Improvement in BDI	4
**Borst et al, 2014**[102]	Men aged 60 or more with total T ≤300 ng/dL (10.4 nM) or bioavailable T ≤70 ng/dL (2.4 nM)	T enanthate 125 (n = 14) or 0 (n = 16) mg/week IM for 23 months. There also were finasteride + T arms that we ignore here.	T-associated reduction of a mean of 0.74 items on the 15-item Geriatric Depression Scale short form	1
**Mirdamadi et al, 2014**[22]	50 males, age 50–70, with CHF	T enanthate 250 mg IM or saline placebo IM every 4 weeks for 12 weeks	No difference between groups in BDI	3
**Hackett et al, 2014**[58]	199 men aged 18–80 with T2DM with total T between 8.1–12 nM (233–346 ng/dL) or total T ≤8.0 nM (231 ng/mL). 9 patients did not complete the study; 4 because of serious adverse events (3 treatment unrelated deaths, 1 prostate cancer in placebo), and 5 withdrew their consent.	Subjects were block randomized to receive testosterone undecanoate 1000 (n = 97) or 0 (n = 102) mg IM at week 0, 6, and 18.	No improvement in Hospital Anxiety and Depression score (HADS) in men with baseline T ≤8.0 nM; ↑HADS-depression score but not HADS-anxiety in men with baseline T 8.1–12 nM	5
**Malkin et al, 2004**[1]	12 men 60.8 ± 4.6 years old (mean ± SD) with CAD and “clinical need for T replacement.” One man failed screening and another withdrew at unspecified point in the study.	100 mg T or placebo IM every 2 weeks for 4 weeks, 1 month washout, then tx switch; randomization by computer; single-blinded	↓BDI score	5
**Menwith Psychiatric Disorders**				
***HIV-Positive***				
**Grinspoon et al, 2000**[129]	52 men, mean age 41.6, with AIDS-associated wasting (weight <90% of ideal or weight loss >10%) and serum free T concentration <12 pg/mL (416 pM); 21 of these men had baseline BDI >18; 13 subjects died or withdrew and were not evaluated.	T 300 mg (n = 21) or placebo (n = 18) IM every 3 weeks for 6 months.	Improved BDI by a mean of 5.8 points; improvement was associated with increased weight. BDI did not change with placebo. Men with BDI >18 at baseline were not analyzed separately.	3
**Rabkin et al, 2000**[35]	72 HIV-positive men, mean age 39, with serum T <17.4 nM (501 ng/dL) with sexual dysfunction and at least one “hypogonadal” mood symptom; 2 dropped out and were excluded from analysis. Among 70 remaining, 26 had MDD, dysthymia, “minor” depression, or MDD in remission.	Randomization in blocks of 4 by computer-generated numbers to T cypionate (n = 38 overall, 26 with depression diagnosis) or placebo (n = 32 overall, 7 with a depression diagnosis) injected biweekly (presumably IM) for 6 weeks; the first T dose was 200 mg, subsequent doses were 400 mg. Open-label phase with T followed the 6-week double-blind study, not summarized here	Clinical Global Impression (CGI) scale response (not defined) in 74% T and 19% placebo (*P* < 0.001). No effect of tx on CGI in subjects with a depression diagnosis (*P* = .08). Improvement in total and vegetative scores on HAM-D, but not the affective scale. No change in the BDI (*P* = .052)	4
**Rabkin et al, 2004**[130]	123 HIV-positive men, mean age 41, with MDD or dysthymia; 33 men dropped out and were retained for ITT analysis.	Randomization in blocks of 6 by computer-generated numbers to T cypionate IM biweekly + daily PO placebo (n = 38), placebo IM biweekly + fluoxetine PO (20–40 mg) daily (n = 46), or placebo IM biweekly + placebo PO daily (n = 39). Initial T dose was 200 mg, subsequent doses were 400 mg. Within tx groups, subjects were also randomized to fluoxetine or placebo daily by mouth. Tx were given for 8 weeks. Eight of the subjects were randomized only to fluoxetine or placebo due to transient unavailability of T.	No difference in response on HAM-D or BDI by ITT and by considering only completers. Improvement in Chalder Fatigue Scale compared to placebo and to fluoxetine	4
***HIV-Negative***				
**Reddy et al, 2000**[121]	22 men ≥65 years old; 8 dropouts at different times were analyzed only for those forms they completed.	T enanthate 200 mg (n = 14) or placebo (n = 8) IM every 2 weeks for a total of 4 doses.	No effect on Short-Form 36 (SF-36) or Psychological General Well-Being	3
**Seidman et al, 2001**[36]**Seidman & Roose, 2006**.[164]	32 men 33–71 years old with MDD (DSM-IV criteria) and serum T ≤350 ng/dL (12.1 nM); 2 dropouts were excluded from analysis.	T enanthate 200 mg (n = 13) or placebo (n = 17) IM weekly for 6 weeks	Improved QoL score. HAM-D and BDI described as improved in both groups; no intergroup difference. No difference between groups in response rate, defined as a ≥50% decrease in the HAM-D or as defined by CGI	4, 4
**Pope et al, 2003**[125]	23 men with tx-refractory MDD (DSM-IV criteria) and serum T ≤350 ng/dL (12.1 nM); 22 randomized (1 man who responded to run-in placebo gel excluded)	T gel 1% (n = 12) or placebo gel (n = 10) for 8 weeks; valid randomization procedure described. T dose reduced if serum T exceeded 1070 ng/dL; sham reductions performed in placebo gel. Usual antidepressants continued during trial	↑Rate of decrease in HAM-D and CGI scores; no difference in rate of change in BDI ↑Response as evaluated by HAM-D but no significant change in CGI or BDI	5
**Kenny et al, 2004**[124]	11 men with MMSE scores of 14–28, cognitive impairment by Dementia Rating Scale, and serum free T <128 ng/dL (4.44 nM); none discontinued.	T enanthate 300 mg (n = 6) or placebo (n = 5) IM every 3 weeks for 9 weeks; testing at 10 weeks. Method of randomization not given.	No effects on behavior with Behave AD or mood with GDS	3
**Cavallini et al, 2004**[48]	150 men 60–74 years old with symptoms of androgen decline, including decreased libido and erectile quality, depressed mood and concentration, irritability, and fatigue; free T <6 pg/mL (21 pM); 20 dropouts excluded	T undecanoate 160 mg/day (n = 40), carnitine (n = 45, not further considered here), or placebo (n = 45) taken PO for 6 months; randomization by color-coded boxes	HAM-D improved at 3 but not at 6 months	4
**Seidman et al, 2005**[127]	26 men, mean age 46.4 years, with MDD partially or nonresponsive to 2 adequate antidepressant trials	T group (n = 13) received IM T enanthate 200 mg/week IM × 2 weeks, then 400 mg, then 400 mg (if responsive) or 600 mg (if not responsive) for the final injection; placebo group (n = 13) received weekly IM saline for 4 weeks. Tx allocation by random number table. Antidepressants continued. Evaluation at 6 weeks	No difference between groups in improvement by HAM-D or BDI	5
**Orengo et al, 2005**[128]	18 men >50 years old with residual depression (≥12 on HAM-D) in spite of at least 6 weeks of antidepressant therapy and serum T ≤350 ng/dL (12.1 nM); 6 men dropped out and were not included in the analysis.	Random number generator used to randomize men to T 1% gel 5 g/day or placebo gel for 12 weeks followed by crossover to the other tx; 5 subjects received placebo first and 7 subjects received T first. Evaluations every 6 weeks	Improvement in HAM-D scores after 12 weeks of T compared to baseline, but no difference between T and placebo. No effect of tx on QoL Satisfaction Questionnaire or POMS	3
**Lu et al, 2006**[42]	18 men with AD; 2 dropouts were excluded from analysis; last observation carried forward for ITT analysis was reported not to change the results.	T gel 75 mg/day (n = 9) or placebo gel (n = 9) for 24 weeks	No tx effect on Neuropsychiatric Inventory, BDI, or self-assessed QoL-AD. Improvement in caregiver-assessed QoL-AD	3
**Ko et al, 2008**[123]	30 schizophrenic men, 20–49 years old, on antipsychotic medication; 4 of these subjects withdrew and were retained for ITT analysis.	T 1% gel 5 g (n = 15) or placebo (n = 15) applied daily for 4 weeks; method of tx assignment not discussed	Improvement in the negative symptom scores on the Positive and Negative Syndrome Scale. No change in the Calgary Depression Scale for Schizophrenia; the authors implied better results for completers, but there was still no significant difference from placebo.	3
**Seidman et al, 2009**[51]	23 men, mean age 50.6 years, with dysthymia and serum T concentration <350 ng/dL (12.1 nM).	T cypionate 200 mg (n = 13) or placebo (n = 10) IM every 10 days for 6 weeks; randomization by computer	Greater improvement in HAM-D and BDI than with placebo. ↑Remission (CGI of 1 or 2 or HAM-D < 8) for 7 men on T (n = 13) compared to 1 man on placebo (n = 10).	5
**Shores et al, 2009**[126]	33 men ≥50 years old (mean age 59 years) with dysthymia or “minor depression” and T concentration ≤280 ng/dL (9.7 nM); 6 subjects discontinued and were included in ITT analysis.	T gel 7.5 g (n = 17) or placebo (n = 16) for 12 weeks; “randomization” by computer using 1:1 ratio	Greater improvement in HAM-D than placebo. ↑Remission (HAM-D ≤7): 9/17 men in T group vs. 3/16 men in placebo group. No effect on Hopkins Symptom Checklist, SF-36, or QoL Enjoyment and Satisfaction Questionnaire.	5
**Pope et al, 2010**[111]	100 men, 30–65 years old, with MDD with partial or no response to antidepressant therapy and serum T ≤350 ng/dL (12.1 nM); 81 men completed at least 4 weeks of tx, 74 men completed the study. ITT analysis used for 95 subjects who had at least 1 post-baseline evaluation.	T gel 5 g/day or placebo for up to 6 weeks (n = 50/group); the dose could be altered based on serum T concentration. “Randomization” by sealed envelope	No effect on HAM-D, CGI, or Montgomery Asberg Depression Rating Scale (MADRS)	5
**Amiaz et al, 2011**[37]	100 depressed men 30–65 years old with MDD (DSM-IV criteria), with a HAM-D score ≥12 in spite of currently taking a serotonergic antidepressant for at least 4 weeks, serum testosterone ≤350 ng/dL (12.1 nM); 37 subjects were not evaluable due to loss of data and withdrawal.	T (n = 31) or placebo (n = 32) gel for 6 weeks; allocation using table of random numbers blocked at 50/group; tx period varied from 15 days to 6 weeks.	Improvement in HAM-D and MADRS	5

#### 3.4.1 Healthy men

Some studies of mood and behavior were designed to evaluate the potential adverse effects of anabolic steroid abuse. For example, men abusing anabolic steroids have been described as having “Roid Rage.” We did not evaluate steroid abuse studies, but we reviewed studies on testosterone preparations and their association with anger, aggression, and other mood alterations. There was little consistency among the studies we reviewed.

Five studies reported treatment-associated increases in anger, aggression, or hostility.[67, 108–111] Only two of these studies had a Jadad score of 4 or 5.[108, 111] One study,[110] with a Jadad score of 3, determined that testosterone gel applied to the skin increased hostility based on evaluations by 2 undergraduate judges of a free-text paragraph written by each subject to describe his mood at the end of treatment. We do not know the reliability of this assessment. Two studies (Jadad score 3 and 5) reported a decrease in anxiety after testosterone treatment.[45, 112]

Seventeen of 29 studies reported no effect of testosterone treatment on personality, psychological well-being, or mood.[22, 24, 38, 59, 63, 85, 104, 113–121] Seven of 17 studies had a Jadad score of 4 or 5. One of these studies could not be evaluated because only a composite score for mood and sexual function was reported.[38] The study that used hostility assessments by undergraduate judges found no change in personality as assessed by the Gough and Heilbrun Adjective Check List.[110] Another study in this group reported that elevation of testosterone serum concentrations above normal using testosterone gel was associated with an increase in selfishness on a computer game that evaluated the willingness to give away small amounts of money.[122] Two additional studies from the same group in non-depressed men with CHF did not show an effect of testosterone on the Beck Depression Inventory (BDI),[20, 27] although the earlier of these studies concluded otherwise based on a finding that was not statistically significant. A study in non-depressed men with metabolic syndrome reported an improvement in the BDI in testosterone-treated compared to placebo-treated subjects.[52] A study found non-depressed men older than 60 years to have a mean 5% decrease in a geriatric depression scale when administered testosterone.[102] This study had a Jadad score of 1. Another study [58] found that testosterone treatment had no effect on the Hospital Anxiety Depression score (HADS) in men with testosterone ≤8.0 nM but improved the depression subset of the HADS in men with testosterone of 8.1–12 nM. Malkin et al.[1] found that 100 mg testosterone every 2 weeks improved the BDI score. This study had a Jadad score of 5.

#### 3.4.2 Men with psychiatric diagnoses

Twelve studies (3 in HIV-positive men) evaluated testosterone supplementation in men with a diagnosis of depression or dysthymia (sometimes also called, “minor depression”), 1 study evaluated the use of testosterone in men with schizophrenia, and 2 studies were conducted in men with Alzheimer disease or cognitive impairment. The study in schizophrenic men used testosterone or placebo gel in addition to whatever treatment the subject was already using.[123] There were improvements in the negative symptom scores on a standardized scale but no change in the Calgary Depression Scale for Schizophrenia. The authors used an intention-to-treat (ITT) analysis and implied that better results were seen among subjects who completed the study; however, there were no significant differences in the depression scores between testosterone and placebo among completers. Two studies in men with cognitive impairment or Alzheimer disease (Jadad score 3) found no effect of treatment on neuropsychiatric symptoms, depression, behavior, or quality of life (QoL).[42, 124] Caregiver-assessed QoL was improved in 1 of these studies.[42]

The response of depression and dysthymia to testosterone was mixed and inconsistent. Among HIV-negative men, four studies (all with a Jadad score of 4 or 5) showed testosterone-associated improvements in standard scoring systems for depression and/or in the proportion of subjects who achieved remission of their psychiatric disorder.[37, 51, 125, 126] Four other studies (2 with a Jadad score of 4 or 5) showed no improvement in depression or dysthymia with testosterone compared to placebo.[36, 111, 127, 128] One study (Jadad score 4) showed a transient improvement in depression and melancholia after 3 months of treatment that was no longer apparent after 6 months of treatment.[48]

Because it has been noted that HIV-positive men can be depressed and “hypogonadal,” 3 studies administered testosterone to HIV-positive men with depression or dysthymia.[35, 129, 130] Two of the studies had a Jadad score of 4[35, 130] and one study had a Jadad score of 3.[129] Testosterone treatment had inconsistent effects on measures of depression; one study showed a 5.8-point improvement in the Beck Depression Inventory (BDI) in men with HIV-associated wasting, although the improvement may have been explained by an increase in weight.[129] Another study showed a testosterone-associated improvement in HIV-positive men overall in the Clinical Global Impression (CGI) scale but not among subjects with a depression diagnosis.[35] This study also showed improvement in the total and vegetative symptom scores of the Hamilton Rating Scale for Depression (HAM-D) but not in the affective scale, and there was no significant change in BDI scores. A subsequent, larger study by the same group showed no difference in response of depression measured by HAM-D or BDI in men given testosterone compared to placebo.[130]

Authors attributed the mixed responses in the literature to the considerable placebo response in most studies and to the possibility of an idiosyncratic response to testosterone, with putative subgroups of responders who were difficult to identify *a priori*.[111, 127] The studies, however, did not show consistent responses in subgroups of men who had low serum testosterone concentrations, depression resistant to standard therapy, or men characterized as middle-aged or elderly. In studies in which serum testosterone concentrations were measured on therapy (both with a Jadad score of 5), response of depression or dysthymia was not consistently associated with serum hormone concentration.

### 3.5 Cognition

Twenty-two studies evaluating the effects of testosterone on cognition are summarized in Table 5. Seventeen focused on men without cognitive impairment and 4 focused on men with cognitive impairment.

**Table 5:** Effects of Testosterone on Cognition.

**Menwithout Cognitive Impairment**				
**Janowsky et al, 1994**[114]	56 healthy men 60–75 years old	15 mg T scrotal (n = 27) or placebo patch (n = 29) 16 hours daily for 3 months; method of randomization not discussed	No effect on verbal memory (California Verbal Learning Test), visual memory (Visual Reproduction subtest of the Wechsler Memory Scale), manual dexterity (Grooved Pegboard Test), cognitive flexibility (Trail-Making Test [TMT]). Improved spatial cognition (revised block design subtest of Wechsler Adult Intelligence Scale [WAIS]), attributed to decreased estradiol	3
**Dobs et al, 1999**[113]	133 men with AIDS age 18–65+ who had lost 5–20% of baseline weight with a morning serum total T ≤400 ng/dL (13.9 nM) or free T ≤16 pg/mL (56 pM); 15 T and 20 placebo did not complete the study.	15 mg T (n = 67) or placebo (n = 66) patch applied 20–24 hours/day to shaved scrotal skin for 12 weeks; assessments at weeks 4, 8, and 12; randomization method not given	No effect on cognitive assessed by Rand HIV, SF-36, or EUROQoL Feeling Thermometer	3
**Janowsky et al, 2000**[135]	19 men 61–75 years old.	T enanthate 150 mg (n = 10) placebo (n = 9) given IM each week for 1 month; randomization method not given	Improved working memory (Subject Ordered Pointing Test)	2
**Wolf et al, 2000**[133]	30 men, mean age 68 years.	T enanthate 250 mg (n = 17) or placebo (n = 13) given IM one time; testing occurred 5 days after injection; method of randomization not given	Poorer performance on verbal fluency test. No tx effect on spatial or verbal memory (immediate or delayed recall), Stroop test, or mental rotation	2
**O’Connor et al, 2001**[134]	20 men age 19–45 years (mean age 28.2 years)	T enanthate 200 mg (n = 15) or placebo (n = 15) IM weekly for 8 weeks; testing at 4 and 8 weeks	Improved verbal fluency (Controlled Oral Word Association Test). Poorer performance on revised block design subtest of the WAIS. No tx effect on cognitive flexibility (TMT), revised vocabulary subtest of the WAIS, visuomotor dexterity and speed (Grooved Pegboard test), or Rey Auditory Verbal Learning Test (RAVLT)	2
**Cherrier et al, 2001**[131] **and 2004**[170]	28 healthy men age 50–80 (mean age 67 years); 3 dropouts were excluded from analysis after randomization.	T enanthate 100 mg (n = 15) or placebo (n = 13) IM every week for 6 weeks followed by 6-week washout. Testing at weeks 0, 3, 6, and 12. Method of randomization not given	Improvement in some, but not all, tests of spatial memory. No effect on attention or verbal fluency; Story-recall ability associated with T and estradiol serum concentrations. Week 12 (washout) results not shown	5, 5
**Cherrier et al, 2005**[136]	60 men age 50–85 years (mean age 65 years); 3 dropouts after randomization excluded from analysis.	T enanthate 100 mg IM each week + daily placebo pill (n = 20) or T enanthate 100 mg IM each week + anastrozole 1 mg PO daily (n = 19); IM or placebo injection every week for 6 weeks + daily placebo pill (n = 21) followed by 6-week washout. Testing at weeks 0, 3, 6, and 12. Method of randomization not given	Improvement in spatial memory with T tx only at the washout visit. Improvement in spatial memory with T + anastrozole at 6 weeks and at the washout visit. Improvement in verbal recall with T tx, not persisting to washout visit. No effects on verbal fluency, attention, or working memory	5
**Lu et al, 2006**[42]	29 healthy men; 7 dropouts were excluded from analysis; last observation carried forward for ITT analysis was reported not to change the results.	T gel 75 mg/day (n = 14) or placebo gel (n = 15) for 24 weeks; method of randomization not given	No tx effect on Alzheimer Disease Assessment Scale-Cognitive Subscale (ADAS-COG), California Verbal Learning Test, revised block design subtest of the WAIS, Judgment of Line Orientation, or Developmental Test of Visual-Motor Integration	3
**Maki et al, 2007**[120]	15 men age 66–86 years, total blood T >240 ng/dL (8.3 nM)	T enanthate 200 mg (n = 9) or placebo (n = 6) IM every other week for 90 days followed by 90-day washout followed by tx switch; tx order determined by coin toss	Poorer performance in verbal memory (California Verbal Learning Test). No tx effect on short-term memory for geometric figures (Benton Visual Retention Test), attention and working memory (Digit Span), working memory and attention (α-Span), manual dexterity (Grooved Pegboard), attention, visuomotor scanning, and cognitive flexibility (TMT)	5
**Cherrier et al, 2007**[140]	57 healthy men age 50–90 years (mean age 67 years).	T enanthate 50, 100, or 300 mg or placebo IM weekly for 6 weeks followed by 6-week washout. Testing at weeks 0, 3, 6, and 12. Randomized using random number generator. Number of subjects in each group not given	Performance by assigned dose group not given. Improved verbal and spatial memory in men whose serum T increased 11–50 nM (318–1446 ng/dL) above baseline (3 men received 50 mg T enanthate, 17 men received 100 mg, and 2 men received 400 mg)	5
**Emmelot-Vonk et al, 2008**[91]	237 healthy men 60–80 years old with T < median; ie, <13.7 nM (395 ng/dL); 30 dropouts, 16 of whom provided some follow-up information	T undecanoate 160 mg (n = 120) or placebo (n = 117) by mouth daily for 6 months; randomization by computer-generated list using blocks of 6	No effect on cognitive function (Benton Judgment of Line Orientation, Digit symbol substitution, Shepard Mental Rotation, RAVLT, TMT)	5
**Sih et al, 1997**[104]	32 men age 51–79 with free blood T <60 ng/dL (2.1 nM); unclear how dropouts after randomization were handled	T cypionate 200 mg (n = 10) or placebo (n = 12) every 14–17 days for 12 months; tx allocation by random number (not otherwise characterized)	No effect on memory (RAVLT+ recall; Rey Visual Design Learning Test + recall; Animal Naming)	4
**Ly et al, 2001**[11]	37 men mean age 68.2 years with plasma T ≤15 nM (432 ng/dL); 4 dropouts were excluded from analysis.	DHT gel 70 mg (n = 18) or placebo (n = 19) applied daily for 3 months; method of randomization not discussed	No effect or possible worsening of MMSE (placebo group had a “minor increase in total score and 3-word recall that was not evidence in DHT-treated participants.” Data were not shown.	5
**Kenny et al, 2002**[137]	67 men age 65–87 years (mean 76 years) with free T <128 ng/L (4.44 nM); 23 dropouts appear excluded from analysis	T 5 mg/d (n = 24) or placebo patch (n = 20)	No tx effect on Digit Span, Digit Symbol, or TMT	4
**Haren et al, 2005**[119]	76 men 60–86 years old, mean age 68.5, with at least 2 symptoms on the ADAM questionnaire and total T >8 nM (231 ng/dL); 6 dropouts analyzed by ITT	T undecanoate 160 mg/day (n = 39) or placebo (n = 37) by mouth for 12 months; dose halved if hematocrit increased above 50%; randomization in blocks of 4 using ADLS	No effect on TMT B, visuospatial block test, or MMSE	5
**Okun et al, 2006**[85]	Men with Parkinson disease and free T <100 pg/mL (347 pM), mean age 68 years	T enanthate 200 mg (n = 15) or placebo (n = 15) IM every 2 weeks for 8 weeks; dose could be adjusted upwards based on free T concentration measured every 2 weeks; method of randomization not given	Improved scores on Hopkins Verbal Learning Test and backward visual span. No effect on Controlled Oral Word Association, revised block design subtest of the WAIS, Mental Rotations Test, digit span, visual span, Subject Ordered Pointing Task, TMT, or Stroop test	4
**Vaughan et al, 2007**[112]	69 men age 65–83 (mean 70.8) with 2 morning serum T concentrations <12.1 nM (350 ng/dL); 23 dropouts excluded from analysis	T enanthate 200 mg IM every 2 weeks + placebo pill (n = 23), T enanthate 200 mg IM every 2 weeks + finasteride 5 mg/day (n = 22), or placebo injection and pill (n = 23), tx for 36 months; block-randomization by computer; possible reduction in T dose if hematocrit >52%; cognitive tests at baseline, 4, and 36 months	Better reversal of digits on forward and backward number sequencing (Digit Span). Better performance on verbal learning (Selective Reminding test). No tx effect on attention (TMT A), executive functioning (TMT B), visuospatial skills (Judgment of Line Orientation), visual memory (Benton Visual Retention test)	5
**Menwith Cognitive Impairment**				
**Tan & Pu, 2003**[138]	10 men age 68–80 (mean 72.4) with a new diagnosis of AD with total T <240 ng/dL (8.3 nM); management of dropouts not discussed	T enanthate 200 mg (n = 5) or placebo (n = 5) IM every two weeks for 12 months; randomization method not given. Testing every 3 months; statistical methods not given	Improved performance on ADAS-COG and MMSE.	2
**Kenny et al, 2004**[124]	11 men age 73–87 with MMSE scores of 14–28, cognitive impairment by Dementia Rating Scale, and serum free T <128 ng/dL (4.44 nM); no subject discontinued	T enanthate 300 mg (n = 6) or placebo (n = 5) IM every 3 weeks for 9 weeks; testing at 10 weeks. Method of randomization not given	No effects on behavior (Behave AD), activities of daily living (Katz ADL), and cognition (Digit Span, Clock face, verbal fluency, TMT B)	3
**Cherrier et al, 2005**[132]	32 men, 63–85 years old, diagnosed with probable AD or mild cognitive impairment; 4 dropouts excluded from analysis	T enanthate 100 mg (n = 19) or placebo (n = 13) IM weekly for 6 weeks followed by 6-week washout. Testing at 3, 6, and 12 weeks. Randomization by random number generator	Improved spatial and verbal memory did not persist during washout period. No effect on verbal fluency or attention	5
**Lu et al, 2006**[42]	18 men with AD; 2 dropouts excluded from analysis; last observation carried forward for ITT analysis reported not to change results	T 75 mg/day (n = 9) or placebo gel (n = 9) for 24 weeks	No tx effect on ADAS-COG, California Verbal Learning Test, revised block design subtest of the WAIS, Judgment of Line Orientation, or Developmental Test of Visual-Motor Integration	3
**Cherrier et al, 2015**[139]	22 men age 60–90 with mild cognitive impairment and total T concentration <300 ng/dL (10.4 nM) and AUA symptom score <19	Transdermal testosterone gel 50 to 100 mg/day, with a target total T level of 500 to 900 ng/dL (n = 10) or placebo gel (n = 12) X 6 months	No significant changes in measures of cognition, mood, or quality of life	5

#### 3.5.1 Men described as normal

Ten studies evaluated the effects of testosterone treatment on cognitive endpoints in healthy men. Spatial cognition/memory was reported to be improved with testosterone supplementation in 3 studies,[114, 131, 132] unchanged in 2 studies,[42, 133] and poorer with supplementation in 1 study.[134] Although 1 study reported improved working memory[135] and 1 study found improved verbal fluency,[134] most other studies found no improvement in verbal fluency, memory, or other cognitive endpoints in healthy men given testosterone.[42, 91, 120, 131, 133, 134, 136] Two of 5 studies that showed improvement and 4 of 7 of the studies that showed no improvement had a Jadad score of 4 or 5.

#### 3.5.2 men described as hypogonadal

Hypogonadal men, variously defined, were found in 1 study to have better verbal learning and reversal of digits on number sequencing with testosterone supplementation,[112] but no effect on the same domain was found in another study.[137] The study showing an advantage used injected testosterone enanthate 200 mg while the negative study used a daily 5 mg patch. Another injection study found no effect of supplementation on memory in hypogonadal men.[104] One study reported a possible disadvantage of treatment with dihydrotestosterone compared to placebo in performance on the Modified Mini-Mental State Examination (MMSE),[11] but data were not shown and the putative difference could not be evaluated. Another study showed no improvement in visuospatial cognition or MMSE with testosterone treatment for 12 months.[119] All studies had Jadad scores of 4 or 5.

#### 3.5.3 Men with cognitive impairment

Treatment of men with suspected or diagnosed Alzheimer disease or cognitive impairment was reported in five studies, two of which had a Jadad score above 3. Although 1 injection study found an improvement on the Alzheimer Disease Assessment Scale-Cognitive Subscale (ADAS-COG),[138] another study using testosterone gel found no effect on the same instrument or on other cognitive function tests.[42] Spatial and verbal memory were improved after 6 weekly injections of testosterone enanthate in 1 study, but the effect did not persist during a 6-week washout period without treatment.[132] A fourth study found no effect of testosterone injections on behavior, activities of daily living (ADLs), or cognition.[124] The fifth study found that transdermal testosterone gel was not associated with statistically significant changes in measures of cognition, mood, or quality of life.[139]

#### 3.5.4 Proposed explanations for inconsistent results

Because study results have been varied and inconsistent, some authors have proposed that testosterone is not the only factor or even the most important factor in cognitive function. Janowsky et al[114] found improved spatial cognition in men treated with scrotal testosterone patches, but there was an imbalance between placebo and testosterone groups in baseline blood concentrations of 17β-estradiol, which these authors attributed to chance. The effect of testosterone and 17β-estradiol on spatial cognition testing was explored using *post-hoc* testing, and the putative testosterone effect on spatial cognition appeared to be associated with suppression of 17β-estradiol by testosterone supplementation rather than a direct effect of testosterone. This study had a Jadad score of 3.

Most authors with an interest in 17β-estradiol have suggested that the effectiveness of testosterone, when it has shown effectiveness, is due to aromatization to 17β-estradiol. Cherrier et al[131] measured testosterone and 17β-estradiol concentrations after injection of testosterone supplements in healthy men and reported that both testosterone and 17β-estradiol concentrations were associated with recall of a test story, but only 17β-estradiol concentrations were associated with performance on the Stroop test. In another study, Cherrier et al reported that only men with an increase in 17β-estradiol concentration after testosterone supplementation showed improvements in verbal memory testing.[136] This study used administration of the aromatase inhibitor anastrozole to differentiate between effects attributable to testosterone and those that might be due to 17β-estradiol. Another study without anastrozole found 17β-estradiol serum concentrations after testosterone therapy to be a significant predictor of performance on verbal memory testing.[140] All three studies had a Jadad score of 5.

It has also been suggested that testosterone supplementation has produced inconsistent results in cognitive function studies because the blood concentrations achieved by supplementation need to be in an optimum range for effectiveness. Under this hypothesis, over-supplementation is as ineffective as under-supplementation. Cherrier et al[140] administered testosterone enanthate IM at 0, 50, 100, or 300 mg weekly and administered tests of verbal and spatial memory. Results were not reported according to treatment group; rather, subjects were divided into those with no, moderate, or large increases in serum testosterone concentrations over baseline. These response categories were defined based on 1 standard deviation above the control response and 1 standard deviation above the response to 100 mg. Subjects with a moderate increase in serum testosterone concentration over baseline (defined as 11–50 nM) performed better on cognitive testing than those with “no increase” (0–10 nM) or a large increase (>51 nM). Seventeen of the 22 men in this moderate-increase group had received testosterone 100 mg/week with the balance evenly divided between the 50 mg and 300 mg doses. The authors explained that they did not use tertiles or quartiles, because using quartiles or tertiles resulted in some placebo patients with significant changes from baseline, raising the question of whether men on placebo with an increase in their serum testosterone concentration were distinguishable on cognitive testing from men who received testosterone supplementation. This study had a Jadad score of 5.

In summary, there is no support for the use of testosterone to enhance cognition in normal or cognitively impaired men.

## 4. Discussion

This systematic review examined published RCTs of testosterone supplementation for cardiovascular disease or surrogates of cardiovascular disease, sexual function, muscle strength, mood, and cognition. The review was limited to published studies in English and to trials indexed before April 9, 2016. The evidence supporting the use of testosterone for preventing or treating cardiovascular disease is inconsistent and, on balance, unconvincing. Some evidence supported an acute and chronic effect of testosterone therapy on increasing time to ST-segment depression, and there is evidence of improvement in some measures of congestive heart failure. Most studies showed no effect of testosterone therapy on inflammatory markers, and the effects on lipids were inconsistent.

Studies that examined clinical effects have not favored testosterone therapy over placebo. Two of 3 studies that assessed angina showed no effect. Three studies from the same group found a benefit for symptoms associated with CHF. One study was stopped early for cardiovascular adverse effects.

Testosterone supplementation did not demonstrate consistent effectiveness for improving sexual function or satisfaction. Testosterone is ineffective in treating ED. Controlled trials were mixed on libido, with more positive than negative studies.

Substantial evidence supports a favorable effect of testosterone treatment on muscle mass in both healthy men and men with HIV, and a majority of studies showed a decrease in fat mass. Testosterone did not affect most measures of muscle strength. While decreasing frailty and increasing strength in older men might be beneficial, testosterone supplementation does not improve physical function in older men.

Most studies on mood-related endpoints found no beneficial effect of testosterone treatment on personality, psychological well-being, or mood. Although 2 studies showed decreased anxiety, treatment of depression showed mixed and inconsistent results. Even if testosterone did benefit mood, social adverse events might ensue; 5 studies noted treatment-related increases in anger, aggression, or hostility. Testosterone did not benefit cognitive impairment or Alzheimer disease; neither did it benefit verbal fluency, memory, or other cognitive endpoints in normal men.

In summary, evidence from RCTs does not support treatment of so-called low-T for improving physical function, sexual function, mood, or cognition. Testosterone increases muscle mass, but not strength, and while some improvement is seen in some surrogate markers of cardiovascular risk, there is little evidence of clinical benefit.

There is conflicting evidence on the association between testosterone supplementation and cardiovascular events. RCTs have reported increased cardiovascular risk with testosterone therapy. One such trial that specifically examined cardiovascular disease and mortality endpoints was stopped early because of an increased risk of cardiovascular events.[17] A meta-analysis of 2994 men in 27 randomized controlled trials through 2012 found that testosterone therapy increased the risk of cardiovascular events (OR, 1.54; 95% CI, 1.09–2.18).[141]

Observational studies examining the effect of testosterone treatment have shown conflicting results on risk. A Veterans Administration study evaluated men who had undergone coronary angiography and had a total testosterone concentration (presumably plasma) less than 300 ng/dL (10.4 nM).[142] Men who were treated with testosterone had an increased risk of all-cause mortality, MI, and stroke compared to men who did not use testosterone (HR, 1.29; 95% CI, 1.05–1.58), based on a mean of 27.5 months of follow-up. Another retrospective cohort study using Veterans Administration data showed a lower rate of all-cause mortality, myocardial infarction, and stroke among testosterone-treated men whose testosterone concentrations “normalized” after treatment.[143] Another observational study of men in a large, integrated health care organization found that death rates were reduced over 3 years, but there was no effect on myocardial infarction or stroke.[144]

A Medicare-based study identified testosterone exposures and MI outcomes using claims data and matched testosterone-treated with untreated subjects using an empirically derived propensity score and found no increased risk.[145] The adjusted HR for testosterone therapy and MI was 0.84 (95% CI 0.69–1.02). Analysis of subjects in the highest quartile propensity score range suggested a protective effect of testosterone treatment, with a HR of 0.69 (95% CI 0.53–0.92). An observational study in men with low testosterone found that treatment was associated with reduced mortality;[146] another in diabetics[147] reported benefit on all-cause mortality but excluded men who had received testosterone for less than one year and excluded deaths occurring before six months. A large cohort study found that myocardial infarction rates were significantly increased within three months of testosterone treatment initiation; testosterone-treated men over 65 experienced double the rate of myocardial infarctions compared to men who did not received testosterone.[148]

Testosterone treatment has been considered for disease prevention because men who are obese, diabetic, hypertensive, or chronically ill have lower plasma concentrations of testosterone.[149] However, the direction of causality is unclear; it is possible that obesity or lack of exercise and chronic disease lower testosterone rather than low testosterone concentrations causing disease. It is also possible that another mechanism both lowers testosterone concentrations and increases the risk of some diseases. Observational studies attributing positive health effects to testosterone may be affected by an increased likelihood of healthier men being prescribed testosterone rather than testosterone improving health.

There are parallels between the recommendation of testosterone and of menopausal hormone therapy in women. Physicians prescribed estrogen and estrogen-progestin preparations to menopausal women to prevent cardiovascular disease because observational studies showed that women who took menopausal hormones had less heart disease than women who did not. RCTs, however, showed that menopausal hormone therapy increased the risk of heart attacks and stroke.[150–153] It is likely that healthier women chose to take menopausal hormone therapy, but menopausal hormone administration did not improve health.

In 2012, sales for testosterone therapies exceeded $2 billion, and sales continue to grow in dozens of countries.[154] To the extent that this increase in use of testosterone supplementation is based on anticipated improvements in cardiovascular health, sexual function, physical functioning, mood, or cognition, we suggest that it might represent therapy without adequate clinical trial support. We identified no population of normal men for whom the benefits of testosterone use outweigh its risk. Given the known risks of testosterone therapy and the lack of evidence for clinical benefits in normal men, we do not think further trials of testosterone are necessary.
